# Distant Cutaneous Metastasis of Chordoma: A Systematic Review and Individual-Participant-Data Meta-Analysis of 52 Cases, with an Illustrative Poorly Differentiated Index Case

**DOI:** 10.3390/cells15151348

**Published:** 2026-07-27

**Authors:** Andreea-Adriana Neamțu, Raul Chioibaș, Alicia Goldenberg, Laura Maghiar, Robert Barna, Andrada Iftode, Titus Grecu, Sorina Taban, Aleodor Andea

**Affiliations:** 1 Department of Toxicology, Faculty of Pharmacy, “Victor Babeș” University of Medicine and Pharmacy Timișoara, Eftimie Murgu Square, No. 2, 300041 Timișoara, Romania; andreea.neamtu@umft.ro (A.-A.N.); andradaiftode@umft.ro (A.I.); 2 Research Centre for Pharmaco-Toxicological Evaluation, Faculty of Pharmacy, “Victor Babeș” University of Medicine and Pharmacy Timișoara, Eftimie Murgu Square, No. 2, 300041 Timișoara, Romania; 3 Department of Pathology, Clinical County Emergency Hospital of Arad, Andrenyi Karoly Str., No. 2–4, 310037 Arad, Romania; 4 Department of Pathology, “Pius Brînzeu” Clinical County Emergency Hospital Timișoara, Liviu Rebreanu Boulevard, No. 156, 300723 Timișoara, Romania; 5 Department of Surgery I, Faculty of Medicine, “Victor Babeș” University of Medicine and Pharmacy Timișoara, Eftimie Murgu Square, No. 2, 300041 Timișoara, Romania; office@medcom.ro; 6 CBS Medcom Hospital Timișoara, Popa Șapcă Str., No. 12, 300047 Timișoara, Romania; 7 Department of Pathology, Roswell Park Comprehensive Cancer Center, Elm and Carlton Streets, Buffalo, NY 14263, USA; aleodor.andea@roswellpark.org; 8 Department of Psycho-Neurosciences and Rehabilitation, Faculty of Medicine and Pharmacy, University of Oradea, Universității Str., No. 1, 410087 Oradea, Romania; 9 Department of Dermatovenerology, Clinical County Emergency Hospital Bihor, Gheorghe Doja Str., No. 65, 410169 Oradea, Romania; 10 Department of Microscopic Morphology—Anatomic Pathology, ANAPATMOL Research Center, Faculty of Medicine, “Victor Babeș” University of Medicine and Pharmacy Timișoara, Eftimie Murgu Square, No. 2, 300041 Timișoara, Romania; robert.barna@umft.ro (R.B.); taban.sorina@umft.ro (S.T.); 11 Department of Plastic Surgery, The Christie NHS Foundation Trust, 550 Wilmslow Road, Manchester M20 4BX, UK; titus.grecu@nhs.net

**Keywords:** chordoma, cutaneous metastasis, poorly differentiated chordoma, brachyury, TBXT, SMARCB1/INI1, physaliphorous cells, differential diagnosis, systematic review, individual-participant-data meta-analysis

## Abstract

**Highlights:**

- First focused synthesis of distant cutaneous chordoma metastasis: 52 cases from 40 reports, pooled in a one-stage individual-participant-data meta-analysis (PRISMA 2020).
- A consistent signature emerges—an older adult (median 59 years) with a sacrococcygeal primary (74%, 95% CI 59–85), male predominance 2.3:1, and a median latency of four years.
- Age sharply stratifies dissemination: patients under 40 reach cutaneous spread far earlier (HR 17.2, 95% CI 4.7–62.9), with enrichment for poorly differentiated/INI1-deficient tumours and multifocal deposits.
- The rarity of confirmatory testing is a diagnostic-era artefact: brachyury use rose significantly over time (ρ = 0.43, *p* = 0.008) while SMARCB1/INI1 remained almost never applied, so the historical literature under-counts poorly differentiated chordoma.
- Nuclear brachyury is the decisive discriminator from mimics and SMARCB1/INI1 resolves the poorly differentiated subtype; we provide explicit exclusion criteria and a stepwise diagnostic algorithm.

**Abstract:**

Chordoma is a rare notochordal malignancy of the axial skeleton, and distant cutaneous metastasis is among its rarest manifestations—an under-recognised mimic of benign and malignant skin tumours that often presents with an incomplete clinical history. Methods: We performed a systematic review and one-stage individual-participant-data (IPD) meta-analysis of all reported cases of distant cutaneous chordoma metastasis, following PRISMA 2020, and complemented it with an illustrative index case of cutaneous poorly differentiated chordoma in a young adult. Missing case-level data were handled by available-case analysis without imputation, with denominators reported throughout. Pooled prevalences were estimated by logistic-normal random-effects models (heterogeneity by I^2^); latency to cutaneous metastasis was modelled as a time-to-event outcome, and brachyury uptake over time by meta-regression. Results: Forty reports (52 cases) were included; individual data were analysable in 43. Median age was 59 years (range, 2 months–88), male-to-female ratio 2.3:1, sacrococcygeal primary 74% (31/42) and median latency 4.0 years (synchronous to 15). Physaliphorous cells, myxoid stroma and vacuolated cytoplasm dominated, yet brachyury and SMARCB1/INI1 were documented in only 6/52 and 1/52 cases. Pooled prevalences were 74% (95% CI 59–85) for a sacrococcygeal primary, 70% (55–82) for male sex and 77% (62–87) for physaliphorous morphology, with no between-report heterogeneity (I^2^ = 0%)—expected when almost every report contributes one patient, so these values consolidate rather than extend the descriptive data. Patients younger than 40 years reached cutaneous metastasis far earlier (hazard ratio 17.2, 95% CI 4.7–62.9; *p* < 0.001), and brachyury use rose across eras (ρ = 0.43, *p* = 0.008). Conclusions: Distant cutaneous chordoma metastasis is a late event that usually signals disseminated disease and a limited life expectancy, commonly months to a few years. Its recognition rests on integrating morphology with nuclear brachyury and, in epithelioid or young presentations, SMARCB1/INI1. We provide a differential-diagnostic framework and algorithm, distinguishing cohort-supported findings from expert interpretation.

## 1. Introduction

Chordoma is a rare, locally aggressive malignant bone tumour showing notochordal differentiation, accounting for approximately 1–4% of primary bone malignancies and arising along the axial skeleton—most often the sacrococcygeum and the skull base [1,2]. Its annual incidence is below one per million [2,3]. Although chordomas grow slowly and are dominated clinically by local recurrence, they metastasize in a substantial minority of patients, with reported distant-metastasis rates ranging widely across series (3–50%) and a predilection for lung, bone, liver and lymph nodes [4,5].

Cutaneous and subcutaneous metastases are exceedingly rare and, when they occur, are easily misattributed to benign nodules or to other malignancies [6]. Cutaneous metastasis is itself an uncommon and deceptive event across tumour types—from visceral carcinomas [7] to bone and soft-tissue sarcomas [8]—so that histopathological examination, rather than clinical impression, is decisive in establishing the correct diagnosis [9], particularly for tumours presenting in unexpected anatomical locations [10]. Such deposits may arise by direct extension or local recurrence, by iatrogenic seeding along surgical tracts, or—for lesions distant from the primary—by haematogenous dissemination, frequently years after the original diagnosis [11,12]. Because this presentation is both uncommon and deceptive, the literature is confined to scattered case reports and small series, and no synthesis has placed the distant cutaneous pattern specifically in focus. Here we keep that focus throughout: we first outline the pathology of chordoma, then systematically review and pool, in a one-stage individual-participant-data meta-analysis, all reported cases of distant cutaneous metastasis; we use an illustrative index case to anchor the synthesis and build an explicit differential-diagnostic framework—with exclusion criteria—culminating in a practical diagnostic algorithm. Throughout, we anchor the diagnosis in the molecular pathogenesis of chordoma—the brachyury (TBXT) notochordal programme and SMARCB1/INI1 loss in the poorly differentiated subtype—and connect these drivers to the emerging, molecularly targeted therapeutic strategies that make their recognition in the skin clinically consequential, so that the cutaneous presentation is read not as a curiosity but as an entry point into the biology and treatment of the disease.

## 2. The Pathology of Chordoma

### 2.1. Origin, Epidemiology and Clinico-Radiological Features

Chordoma recapitulates the embryonic notochord and arises from notochordal remnants retained within the axial skeleton [1,13]. The great majority are sporadic, although rare familial cases and an association with tuberous sclerosis in children are recognised [14]. Tumours present most commonly between the fourth and sixth decades, with a male predominance of roughly 2:1 in sacral and vertebral tumours [1,2]. On magnetic resonance imaging, chordomas are lobulated, lytic, destructive midline lesions that enhance after contrast; grossly they are lobulated, gelatinous masses that destroy bone and extend into adjacent soft tissue [1,15]. High-resolution cross-sectional imaging further aids characterisation and localisation of both primary and metastatic deposits [16].

### 2.2. Histopathology and the Notochordal Phenotype

At low power, conventional chordoma shows a lobular architecture with fibrous bands separating lobules. Tumour cells are arranged in cords or ribbons within a myxoid matrix; they are large, with clear-to-eosinophilic, characteristically vacuolated or “bubbly” cytoplasm—the *physaliphorous* cell that defines the tumour. Prominent nucleoli, anisokaryosis and nuclear pseudoinclusions may be seen, but mitoses and apoptotic bodies are scarce or absent in conventional tumours [1,17].

The immunophenotype reflects a dual epithelial–mesenchymal differentiation: chordomas co-express cytokeratins (CK8/18/19), EMA, S100 protein and vimentin, while being negative for CK7 and CK20 [17,18]. The diagnostic hallmark is nuclear expression of brachyury (the T-box transcription factor encoded by TBXT), a highly sensitive and specific marker of notochordal differentiation that is essentially absent from the principal histological mimics [13,19,20]. Brachyury is not merely a marker but a driver: germline tandem duplication of TBXT underlies familial chordoma, the common rs2305089 single-nucleotide polymorphism is carried by the overwhelming majority of patients, and somatic copy-number gains of TBXT occur in sporadic tumours; silencing brachyury arrests chordoma cell growth [14,21]. No recurrent diagnostic molecular alteration defines conventional chordoma beyond this notochordal programme, although PI3K-pathway mutations have been described [21]. Whole-genome and targeted analyses nonetheless portray chordoma as a comparatively quiet genome punctuated by recurrent, therapeutically relevant lesions: activation of the PI3K–AKT–mTOR axis (frequently through PTEN loss), homozygous deletion of CDKN2A/2B as one of the commonest and prognostically adverse copy-number events, and inactivating alterations of the SWI/SNF chromatin-remodelling complex (PBRM1, SETD2, ARID1A) together with the LYST alterations identified by sequencing [21,22,23,24]. Receptor tyrosine kinases—PDGFRB, KIT and EGFR—are commonly expressed and phosphorylated, providing the rationale for the clinical activity of imatinib and EGFR-directed agents [25,26]. The poorly differentiated subtype is set apart molecularly by biallelic SMARCB1 (INI1) inactivation, and DNA-methylation profiling now offers an orthogonal means of confirming notochordal identity and of resolving immune-defined subgroups of relevance to emerging immunotherapy [27,28,29,30]. This reliance on nuclear brachyury and, in the poorly differentiated subtype, SMARCB1/INI1 is consonant with the broader dependence of diagnostic and screening pathology on targeted immunohistochemical panels [31].

### 2.3. Histological Subtypes and Their Diagnostic Work-Up

The current WHO classification recognizes conventional chordoma (including the chondroid variant), poorly differentiated chordoma and dedifferentiated chordoma [1]. Their features and ancillary work-up are summarized in Table 1.

#### 2.3.1. Conventional and Chondroid Chordoma

Conventional chordoma is the prototype described above. The chondroid variant contains an extracellular matrix mimicking hyaline cartilage and is the principal source of confusion with chondrosarcoma; co-expression of cytokeratin/EMA and brachyury resolves the distinction, since chondrosarcoma is negative for both [1,18].

#### 2.3.2. Poorly Differentiated Chordoma

Poorly differentiated chordoma is very rare and occurs disproportionately in children and young adults. It is composed of sheets or nests of epithelioid cells with eosinophilic cytoplasm and scattered intracytoplasmic vacuoles; nuclei are round-to-ovoid and vesicular with mild-to-moderate pleomorphism, mitoses are numerous, and focal rhabdoid morphology is common, while classic physaliphorous cells are often absent [27,28]. The defining feature is loss of SMARCB1 (INI1) expression by immunohistochemistry, with homozygous *SMARCB1* deletion demonstrable by FISH; brachyury and cytokeratin remain positive [27,28]. This subtype behaves aggressively and is the histological pattern of the present index case.

#### 2.3.3. Dedifferentiated Chordoma

Dedifferentiated chordoma comprises under 1% of cases and is biphasic, juxtaposing conventional chordoma with a high-grade sarcoma. Brachyury and cytokeratin are preserved in the conventional component but lost in the dedifferentiated sarcomatous component, which may also lose EMA and S100. Together with poorly differentiated chordoma, it carries the worst prognosis [1,32].

### 2.4. WHO Diagnostic Criteria

In practice, a confident diagnosis rests on four pillars [1]: (i) a midline axial bone tumour; (ii) lobules of cohesive, physaliphorous cells in a myxoid or chondroid matrix; (iii) brachyury immunopositivity; and (iv)—for epithelioid/solid forms—loss of SMARCB1 (INI1) to confirm poorly differentiated chordoma. The diagnostic challenge addressed in this review arises when a lesion is encountered outside its expected axial location—in the skin—so that pillar (i) is absent and the history is often unavailable.

## 3. Materials and Methods

### 3.1. Search Strategy and Selection

The systematic review was conducted and reported in full compliance with the PRISMA 2020 statement [33], and the completed PRISMA 2020 checklist is provided as Supplementary Material (Table S3); a protocol was not prospectively registered. PubMed/MEDLINE was searched from inception to June 2025, combining controlled vocabulary (MeSH) and free-text terms for chordoma with metastasis and cutaneous/skin/subcutaneous/soft-tissue qualifiers (e.g., “chordoma” AND (“cutaneous” OR “skin” OR “subcutaneous” OR “soft tissue”) AND (“metastasis” OR “metastatic”)); the reference lists of all eligible articles and relevant reviews were additionally hand-screened to capture older reports not indexed under these terms, an approach commonly used to consolidate sparse case-based literature [34]. Eligibility criteria were defined a priori. We included any peer-reviewed report—case report or case series—of histologically confirmed chordoma with distant cutaneous or subcutaneous metastasis, defined as a dermal/subcutaneous deposit anatomically remote from the primary axial tumour and not explicable by contiguous local extension. We excluded (i) purely local extension, satellite lesions or recurrence within the operative bed contiguous with the primary; (ii) non-cutaneous metastases; (iii) reports lacking histological confirmation of chordoma; and (iv) reports from which case-level data could not be reliably extracted. Two reviewers independently screened titles/abstracts and then full texts, with disagreements resolved by consensus. Because the same patients can appear in more than one publication, potential duplicate and overlapping reports were identified by cross-checking author, institution, sex, age, primary site, treatment dates and lesion description; where overlap was found the most complete or original report was retained and the duplicate removed, so that each patient contributed only once (for example, patients of the Su and Gagne–Su institutional experience were reconciled to avoid double counting). Reports were retained principally in English to permit reliable full-text verification and accurate case-level extraction without translation; where a non-English report carried an English-language abstract or translation sufficient for unambiguous extraction it was included (as for one French-language report [35]), and the five records excluded for language provided no extractable case-level data. This predominantly English-language restriction is acknowledged as a source of language bias in the limitations.

### 3.2. Data Extraction and Individual-Participant-Data Synthesis

Because the evidence comprises case reports and small series, the individual patient—rather than the study—was the natural unit of synthesis; we therefore extracted, harmonised and combined case-level data in a one-stage individual-participant-data (IPD) meta-analysis rather than pooling study-level effect estimates, which are undefined when most “studies” contribute a single patient [36,37]. As individual data were obtained from published reports rather than supplied by the original investigators, the work follows the PRISMA 2020 statement [33], with the PRISMA-IPD extension used as additional guidance where applicable [36]. Two reviewers independently extracted age, sex, primary site, subtype, latency, cutaneous site, lesion pattern, associated metastases, morphology, immunohistochemistry and molecular results into a piloted, standardised form, resolving discrepancies against the source text. Missing data were frequent and were handled by available-case (complete-case, per-variable) analysis: no values were imputed, and for every variable the denominator was the number of cases in which that item was explicitly reported, which is stated alongside each estimate (n/N). A feature recorded as “not performed” (e.g., an immunostain that was not done) was distinguished from one “not reported”, and an unstated marker was never assumed to be negative; consequently, denominators differ across variables and the low absolute counts for brachyury and SMARCB1/INI1 reflect how often these tests were applied, not negativity. Continuous variables are summarised as medians with ranges and categorical variables as frequencies with explicit denominators.

### 3.3. Statistical Analysis

Beyond description, we tested pre-specified, clinically motivated hypotheses. Continuous variables were compared with the Mann–Whitney U test and associations between continuous variables assessed by Spearman’s rank correlation; categorical associations used Fisher’s exact test. The interval from primary diagnosis to cutaneous metastasis (“latency”) was additionally treated as a time-to-event variable and displayed by the Kaplan–Meier method, with groups compared by the log-rank test; because every patient by definition experienced the event, these curves describe the cumulative timing of cutaneous spread rather than survival. For the meta-analytic synthesis, the pooled prevalence of each cardinal clinico-pathological feature was estimated across reports by random-effects models. Because most reports contributed a single patient, a logistic-normal (random-intercept) model was adopted as the primary estimator, with the Freeman–Tukey double-arcsine transformation applied as a sensitivity analysis while acknowledging its recognised instability for sparse data [38]. Between-report heterogeneity was quantified by Cochran’s Q, the I^2^ statistic and the between-study variance τ^2^, and every pooled proportion is reported with a 95% confidence interval [39,40]. The latency to cutaneous metastasis was analysed within a one-stage IPD framework using a Cox proportional-hazards model with age group (<40 versus ≥40 years) as covariate, yielding a hazard ratio for earlier cutaneous spread with robust (sandwich) standard errors [37]. The temporal evolution of confirmatory immunohistochemistry was examined by meta-regression of brachyury use on publication year (Spearman correlation and logistic regression). All tests were two-sided with α = 0.05; given the modest sample and exploratory intent, no correction for multiplicity was applied and results are interpreted as hypothesis-generating. Analyses used Python (version 3.14.0, SciPy, statsmodels and lifelines). The choice of a one-stage IPD framework, rather than a two-stage study-level meta-analysis, was dictated by the data structure: study-specific proportions and hazard ratios are not estimable when the majority of reports contribute a single patient, so case-level modelling is the only coherent option. For the same reason the pooled prevalences are best understood as a formal, variance-weighted consolidation of the case-level proportions rather than as evidence of consistency across independent studies: with predominantly single-patient reports the between-report variance τ^2^ is not estimable, I^2^ is structurally 0%, and the confidence intervals reflect sampling uncertainty in the aggregate cohort rather than heterogeneity between studies. The logistic-normal (random-intercept) model was preferred as the primary estimator because it accommodates single-event reports and small denominators without the boundary instability that the Freeman–Tukey double-arcsine transformation exhibits in sparse data [38], and robust (sandwich) standard errors were used in the Cox model to acknowledge clustering within the few multi-patient series. These estimates are accordingly interpreted as hypothesis-generating.

### 3.4. Risk-of-Bias Assessment

Because the eligible studies comprised both single-patient case reports and a minority of multi-patient case series, methodological quality was appraised with two design-appropriate Joanna Briggs Institute (JBI) instruments, the JBI Critical Appraisal Checklist for Case Reports (eight items) for the single-patient reports, and the JBI Critical Appraisal Checklist for Case Series (ten items) for the multi-patient series, with the Murad tool as a complementary framework [41,42]. Two reviewers independently appraised every included report, with disagreements resolved by discussion. All 40 reports were assessed: the 35 single-patient reports with the case-report checklist, the five series—Su 1993, Chambers 1979, Vogin 2016, Kishimoto 2012 and Dimopoulos 2023—with the case-series checklist (Supplementary Table S2), and three reference-screened reports lacking retrievable full text rated unclear throughout. Because the ratings were initially anchored in the harmonised case-level dataset, items relating to treatment, post-intervention course and adverse events were frequently scored “unclear,” reflecting the diagnostic rather than therapeutic focus of the source literature; the appraisal was cross-checked against the primary full texts where these were retrievable, and unresolved items are reported transparently.

## 4. Results

### 4.1. Study Selection

**Figure 1:**
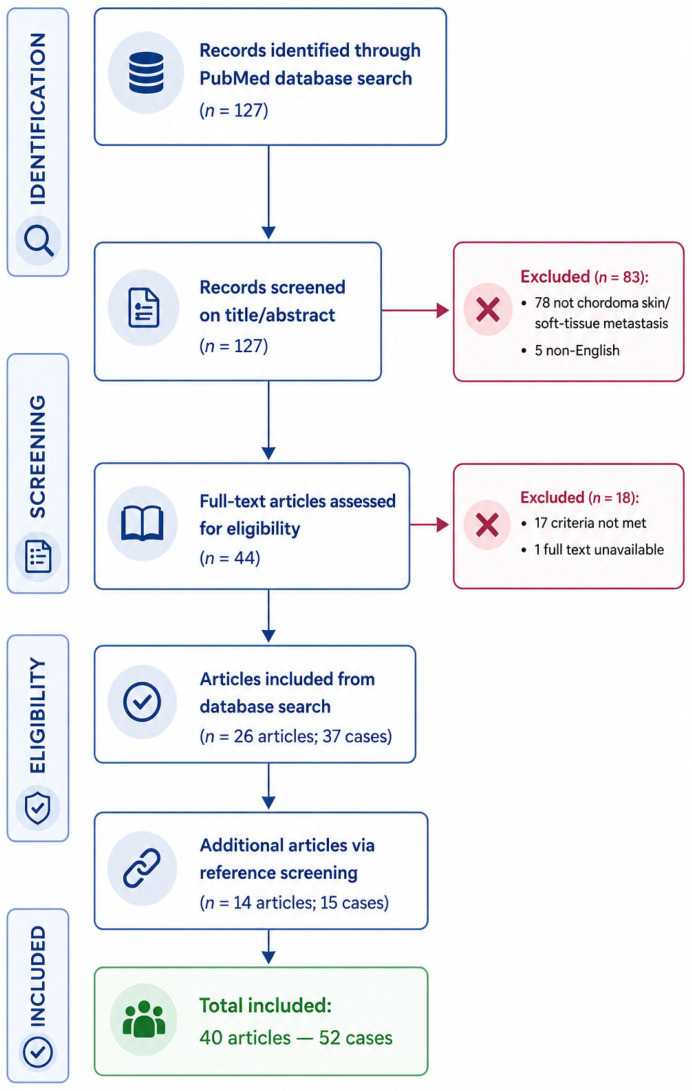
PRISMA flow diagram of study selection.

From 127 records, 83 were excluded at screening (78 unrelated; 5 non-English); 44 underwent full-text review and 18 were excluded. Twenty-six articles (37 cases) were included, with a further 14 articles (15 cases) added by reference screening, yielding 40 reports and 52 cases (Figure 1) [35,43,44,45,46,47,48,49,50,51,52,53,54,55,56,57,58,59,60,61,62,63,64,65,66,67,68,69,70,71,72,73,74,75,76,77,78,79,80,81]. Individual patient data were analysable in 43 of the 52 cases (43/52).

### 4.2. Demographics and Primary Tumour

**Figure 2:**
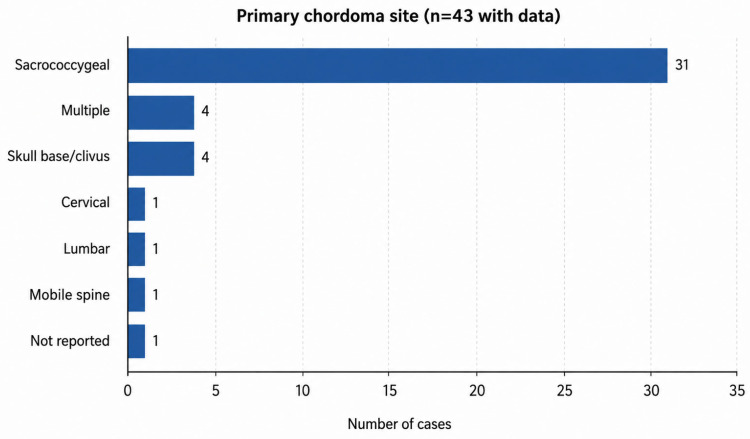
Primary chordoma site among cases with reported data (n = 42).

Median age at cutaneous metastasis was 59 years (range, 2–88 months; n = 40), with a single paediatric case (a congenital, tuberous sclerosis-associated tumour). A male predominance was evident (28 males, 12 females; 2.3:1). The primary was sacrococcygeal in 74% (31/42), with skull base/clival and multiple-site primaries in 4/42 each (Figure 2). Subtype was unspecified in most historical reports (33), with conventional/chondroid in eight and dedifferentiated in two.

### 4.3. Distribution of Cutaneous Metastases and Evidence for Haematogenous Spread

**Figure 3:**
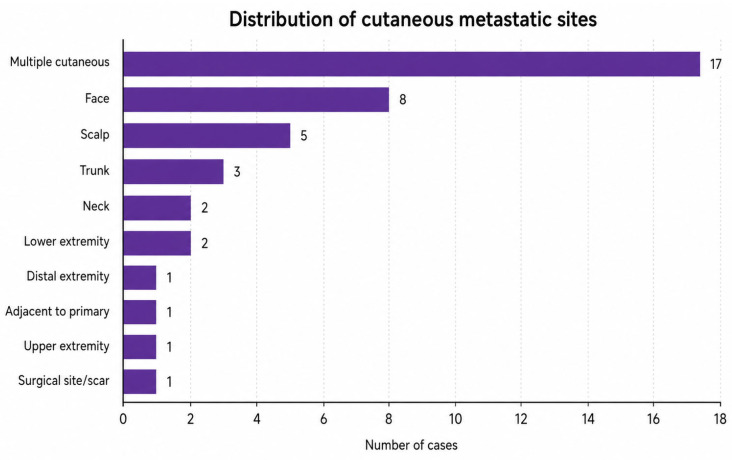
Distribution of cutaneous/subcutaneous metastatic sites (categories not mutually exclusive).

Involvement of multiple cutaneous sites was most frequent (17), followed by the face (8), scalp (5), trunk (3), neck (2) and extremities (Figure 3). The routes of cutaneous spread are heterogeneous. A subset arose within scars or surgical fields, consistent with iatrogenic seeding along instrumented tracts, and a further subset—large sacral tumours ulcerating through the overlying skin—reflected direct local extension rather than true metastasis. Beyond these, however, cross-tabulating primary against cutaneous site was informative: of sacrococcygeal primaries with a localisable skin deposit, nine metastasised to the head and neck and several more to the upper trunk—locations that cannot be reached by contiguous or track-related extension from the pelvis. The most parsimonious explanation for this anatomical mismatch is haematogenous dissemination, and we therefore infer that distant (as distinct from local or iatrogenic) cutaneous chordoma metastasis is predominantly blood-borne. This remains an inference from anatomical distribution rather than from direct vascular or molecular evidence, and iatrogenic seeding and local extension undoubtedly account for a proportion of cutaneous deposits—particularly those adjacent to the primary or to surgical scars—so the three mechanisms are best regarded as coexisting, with haematogenous spread predominating among anatomically remote lesions. Concurrent visceral disease was frequent (lung 14, bone 9, liver 9, lymph node 5) [82], and multiple cutaneous lesions tended to accompany visceral spread more often than solitary lesions (58% vs. 33%; Fisher *p* = 0.20), consistent with a disseminated, blood-borne phenotype.

### 4.4. Latency and Its Determinants: A Distinct Young, Rapidly Disseminating Subgroup

**Figure 4:**
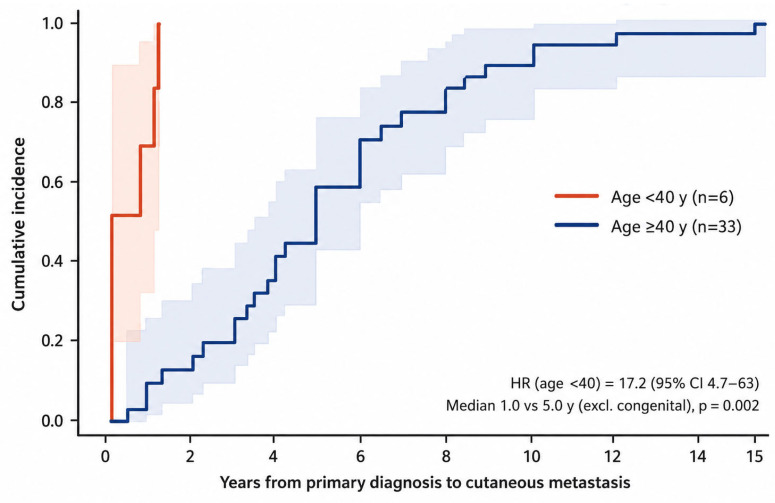
Cumulative time from primary diagnosis to cutaneous metastasis, stratified by age group (Kaplan–Meier). Patients < 40 years reached cutaneous metastasis substantially earlier than those ≥ 40 years.

Overall, the latency from primary diagnosis to cutaneous metastasis was long and right-skewed (median 4.0 years; IQR 1.5–6.0; range synchronous to 15; n = 41). This aggregate, however, conceals a strong age effect. Patients younger than 40 developed cutaneous metastasis far sooner than older patients—median 1.0 versus 5.0 years once the single congenital, tuberous-sclerosis-associated case—whose disease was synchronous at birth—was set aside (Mann–Whitney *p* = 0.002); the difference remained significant when that case was included (*p* = 0.001), and it was reflected in a one-stage IPD Cox hazard ratio for earlier cutaneous spread of 17.2 (95% CI 4.7–62.9; Figure 4) and a continuous age–latency gradient (Spearman ρ = 0.30, *p* = 0.06). The young subgroup was also enriched for aggressive biology: poorly differentiated, dedifferentiated or SMARCB1/INI1-deficient tumours occurred in 2/6 patients under 40 versus 0/34 older patients (Fisher *p* = 0.019), and four of six presented with multiple rather than solitary deposits. Lesion multiplicity itself tracked with shorter latency (median 3.0 vs. 5.0 years for solitary; *p* = 0.054). Taken together, these data delineate a clinically coherent subgroup—young patients with poorly differentiated tumours that disseminate to skin early and multifocally—of which the present index case is a prototypical example.

### 4.5. Evolution of the Diagnostic Work-Up over Time

**Figure 5:**
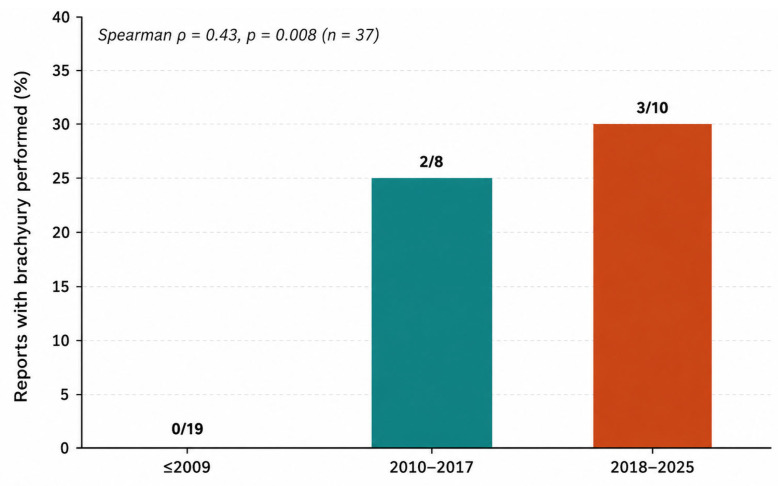
Brachyury immunohistochemistry use across publication periods. Brachyury uptake rose significantly over time (Spearman ρ = 0.43, *p* = 0.008); SMARCB1/INI1 remained almost never applied (≤1 per decade) and is therefore not plotted.

The apparent rarity of confirmatory testing in the literature is largely an artefact of diagnostic era rather than of clinical neglect. Among reports with a recorded publication year, brachyury was performed in none of the reports published up to 2009 (0/19), in two of eight reports during 2010–2017 and in three of 10 during 2018–2025; this upward trend was significant (Spearman ρ = 0.43 between publication year and brachyury use, *p* = 0.008; Figure 5). SMARCB1/INI1, by contrast, was assessed in essentially no cases in any period (≤1/decade), despite its decisive role in the very poorly differentiated tumours that dominate the young subgroup above. The reporting of histological subtype shows the same generational gap, with “unspecified” accounting for most pre-2010 cases. The corollary is important: the historical record systematically under-ascertains poorly differentiated chordoma, and contemporary practice has corrected this only for brachyury, not yet for INI1.

### 4.6. Histology and Immunophenotype Across the Cohort

**Figure 6:**
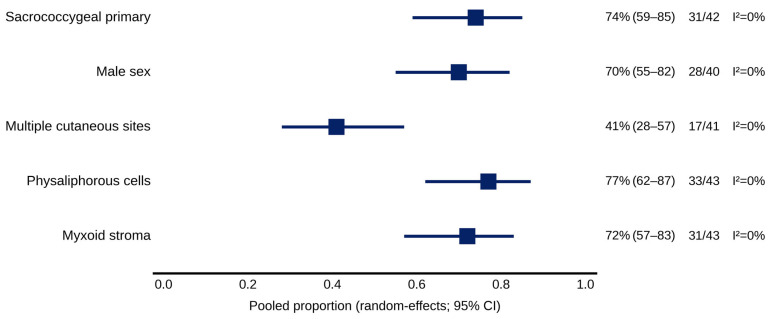
Random-effects pooled prevalence (with 95% confidence intervals) of cardinal clinico-pathological features across the included reports. Between-report heterogeneity was undetectable (I^2^ = 0%) for every outcome, reflecting the predominance of single-patient reports.

Metastatic deposits recapitulated the primary: physaliphorous cells (33), myxoid stroma (31), vacuolated cytoplasm (29), cords/nests (25) and lobular architecture (24) predominated. Among reported immunostains, EMA (24), cytokeratin (20), S100 (19) and vimentin (11) led, reflecting the dual epithelial–mesenchymal phenotype. The low absolute counts for brachyury (6/52) and SMARCB1/INI1 (1/52) should be read in light of the temporal trend in Section 4.5—that is, as a function of how often the markers were applied—rather than as evidence that they are unhelpful (Table 2 and Table 3). Pooling the cardinal features across reports in a random-effects model confirmed their consistency: the pooled prevalence was 74% (95% CI 59–85) for a sacrococcygeal primary, 70% (55–82) for male sex, 77% (62–87) for physaliphorous morphology, 72% (57–83) for myxoid stroma and 41% (28–57) for multiple-site cutaneous involvement, with no detectable between-report heterogeneity (I^2^ = 0% for every outcome; Figure 6). The uniformly null heterogeneity is expected given that almost every report contributed a single patient, so these pooled estimates coincide with the aggregate proportions and are best read as a quantified consolidation of the descriptive findings rather than as evidence of variation between studies.

### 4.7. Pooled Estimates and Risk of Bias Across Reports

**Figure 7:**
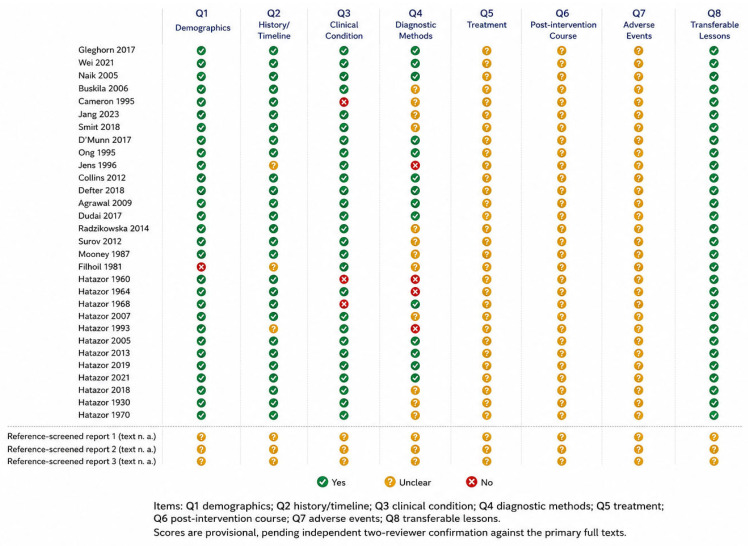
JBI Critical Appraisal Checklist for Case Reports applied to the 35 single-patient case reports. The five multi-patient series were appraised separately with the JBI Critical Appraisal Checklist for Case Series (Supplementary Table S2); three reference-screened reports lacked retrievable full text and were rated unclear throughout [35,43,44,45,46,47,48,49,50,51,52,53,54,55,56,57,58,59,60,61,62,63,64,65,66,67,68,69,70,71,72,73,74,75,76,77,78,79,80,81]. Items: Q1 demographics; Q2 history/timeline; Q3 clinical condition; Q4 diagnostic methods; Q5 treatment; Q6 post-intervention course; Q7 adverse events; Q8 transferable lessons. Scores are provisional, pending independent two-reviewer confirmation against the primary full texts.

The random-effects pooled prevalences of the cardinal clinico-pathological features are shown in Figure 6; with single-patient reports dominating the evidence, between-report heterogeneity was nil (I^2^ = 0%) and the pooled values reproduce the aggregate proportions. Methodological quality, appraised with the design-appropriate JBI checklists (the Case Reports checklist for the 35 single-patient reports, Figure 7; the Case Series checklist for the five multi-patient series, Supplementary Table S2), was generally satisfactory for the items that the diagnostic literature reliably documents—patient demographics, clinical history and timeline, condition at presentation, and the diagnostic work-up—whereas items addressing treatment, post-intervention course and adverse events were frequently rated unclear, mirroring the diagnostic rather than therapeutic orientation of the source material. No report was excluded on quality grounds, and the provisional appraisal is offered for verification against the primary full texts.

## 5. Differential Diagnosis of Distant Cutaneous Metastasis of Chordoma

**Figure 8:**
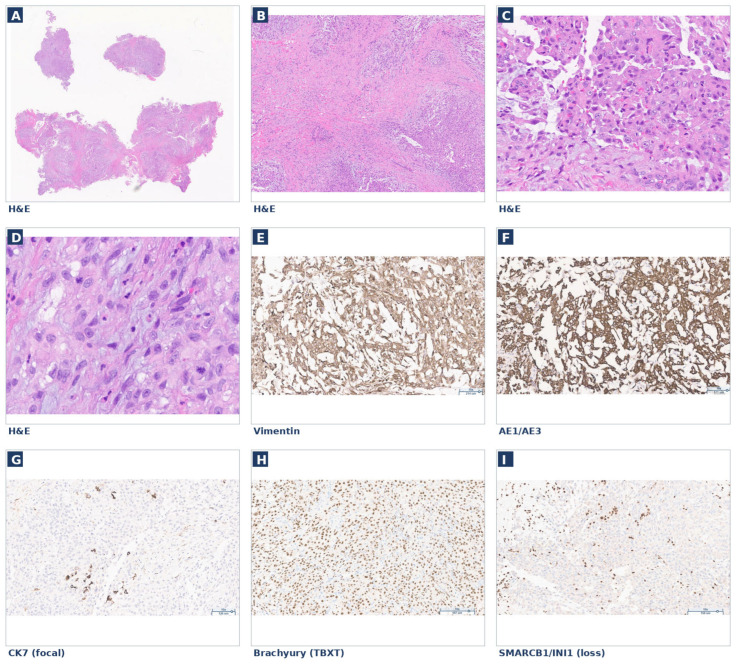
Illustrative index case of distant cutaneous metastasis of poorly differentiated chordoma (right-temporal deposit). A 21-year-old woman presented with a 6-week history of a rapidly growing, occasionally painful, skin-coloured nodule (≈1 cm) in the right temporal region; she reported a separate benign facial skin lesion and a cervical-spine tumour diagnosed at age 14 and reportedly in remission for five years, although the original slides and pathology report were unavailable for review. Computed tomography showed an ovoid soft-tissue density in the right periorbital/temporal subcutis without bone involvement, raising suspicion of recurrence or metastasis; excisional biopsy yielded friable tan-to-white fragments (up to 0.9 cm). (**A**–**D**) Haematoxylin–eosin: sheets and nests of epithelioid cells with eosinophilic, vacuolated cytoplasm (**A**,**B**), vesicular nuclei with numerous mitoses (**C**) and focal rhabdoid morphology (**D**). (**E**–**G**) Dual epithelial–mesenchymal immunophenotype: vimentin (**E**), AE1/AE3 (**F**) and focal CK7 (**G**), with negativity for p40, CK20, SOX10, S100, SMA, CD45 and ERG and no rearrangement on a sarcoma fusion panel. On morphology alone the differential included epithelioid sarcoma, metastatic carcinoma with rhabdoid features, melanoma and other SMARCB1-deficient tumours; re-examination of the limited history (a prior cervical “tumour”) prompted notochordal markers. (**H**,**I**) Strong nuclear brachyury/TBXT (**H**) with loss of SMARCB1/INI1 (**I**) confirmed metastatic poorly differentiated chordoma [1,28]. Integrating morphology, immunophenotype and history yielded the diagnosis. Original magnifications vary by panel.

When a notochordal tumour is encountered in the skin, the absence of an obvious axial mass and the small biopsy sample widen the differential considerably. The unifying principle is that nuclear brachyury is retained in chordoma—including its poorly differentiated and metastatic forms—but is absent in essentially all the mimics [13,19,20]; SMARCB1/INI1 then separates poorly differentiated chordoma from the other INI1-deficient tumours. The entities below, with explicit exclusion criteria, are summarised in Table 4. Throughout this framework we distinguish inferences supported by the pooled cohort from recommendations that rest on expert interpretation and external evidence. The pooled cohort constrains the morphological and demographic profile of cutaneous chordoma, but it can say little about the discriminatory value of individual immunomarkers in the skin: brachyury was recorded in only 6/52 cases and SMARCB1/INI1 in a single case, so their role in the algorithm below is grounded in the primary-tumour and reference literature [13,19,20,27,28], not in these metastatic data. The exclusion criteria are accordingly best read as an evidence-informed expert synthesis rather than as cohort-derived thresholds. The index case (Figure 8) exemplifies this diagnostic challenge.

### 5.1. Benign Notochordal Cell Tumour (BNCT)

BNCT is a benign notochordal lesion occupying the same axial sites and sharing vacuolated/physaliphorous cytology and is therefore the closest morphological relative. It is excluded as a diagnosis for a skin lesion on principle—BNCT is strictly intraosseous and lacks lobular architecture, a myxoid matrix, fibrous septa, conspicuous mitoses and any extraosseous soft-tissue component; radiologically it is sclerotic and non-enhancing, whereas chordoma is lytic, enhancing and destructive [83,84,85]. Both are brachyury-positive, so the distinction is architectural and radiological rather than immunohistochemical.

### 5.2. Chondrosarcoma (And the Chondroid–Chordoma Overlap)

Chondrosarcoma is a malignant cartilage-matrix-producing tumour that overlaps with the chondroid variant of chordoma, especially at the skull base. The decisive exclusion criteria are immunophenotypic and molecular: chondrosarcoma is positive for S100 but negative for cytokeratin/EMA and brachyury, and harbours *IDH1* or *IDH2* mutations, which are not found in chordoma [18,86,87]. Demonstrating epithelial markers and brachyury—or, conversely, an IDH mutation—reliably separates the two.

### 5.3. Atypical Teratoid/Rhabdoid Tumour (AT/RT)

AT/RT is a highly malignant tumour of infants and young children that can mimic poorly differentiated, skull-base chordoma, sharing epithelioid/rhabdoid cytology and—critically—loss of SMARCB1 (INI1). Rhabdoid cells express EMA, SMA and vimentin, often with GFAP, cytokeratins and synaptophysin. The single exclusion criterion that resolves this potentially dangerous overlap is brachyury, which is negative in AT/RT and positive in chordoma; DNA-methylation profiling provides orthogonal separation when needed [1,28].

### 5.4. Myoepithelial Tumours

Myoepithelioma, myoepithelial carcinoma and mixed tumour of soft tissue or bone show trabecular, reticular, nested or solid growth of spindled-to-epithelioid cells in a frequently myxoid stroma, and may co-express S100, EMA, cytokeratin and GFAP, closely imitating chordoma. They are excluded by a negative brachyury and by the presence of *EWSR1*, *FUS* or *PLAG1* rearrangements (with ductal differentiation in mixed tumours) [1,17].

### 5.5. Metastatic Carcinoma

Metastatic carcinoma—particularly mucinous adenocarcinoma and renal cell carcinoma—enters the differential through epithelioid, vacuolated or mucinous morphology and cytokeratin positivity. Carcinomas are excluded by organ-specific markers (TTF-1/napsin-A for lung; PAX8/carbonic anhydrase IX for renal; GATA3/GCDFP-15/mammaglobin for breast; PSA/NKX3.1 for prostate) together with negative S100 and brachyury, and by the absence of a lobulated, myxoid notochordal architecture; cross-sectional imaging of chest, abdomen and pelvis supports the search for a primary [17,20].

### 5.6. Melanoma, Epithelioid Sarcoma and Other Cutaneous Mimics

In the skin specifically, amelanotic or balloon-cell melanoma (S100-positive, vacuolated) is excluded by melanocytic markers (SOX10, HMB45, Melan-A) with negative keratin and brachyury, while epithelioid sarcoma—keratin-positive with SMARCB1/INI1 loss—is separated, like AT/RT, by its negative brachyury [88,89]. Chordoid meningioma and myxopapillary ependymoma are distinguished by EMA-only and GFAP positivity respectively, both brachyury-negative (Table 4).

### 5.7. A Practical Diagnostic Algorithm

**Figure 9:**
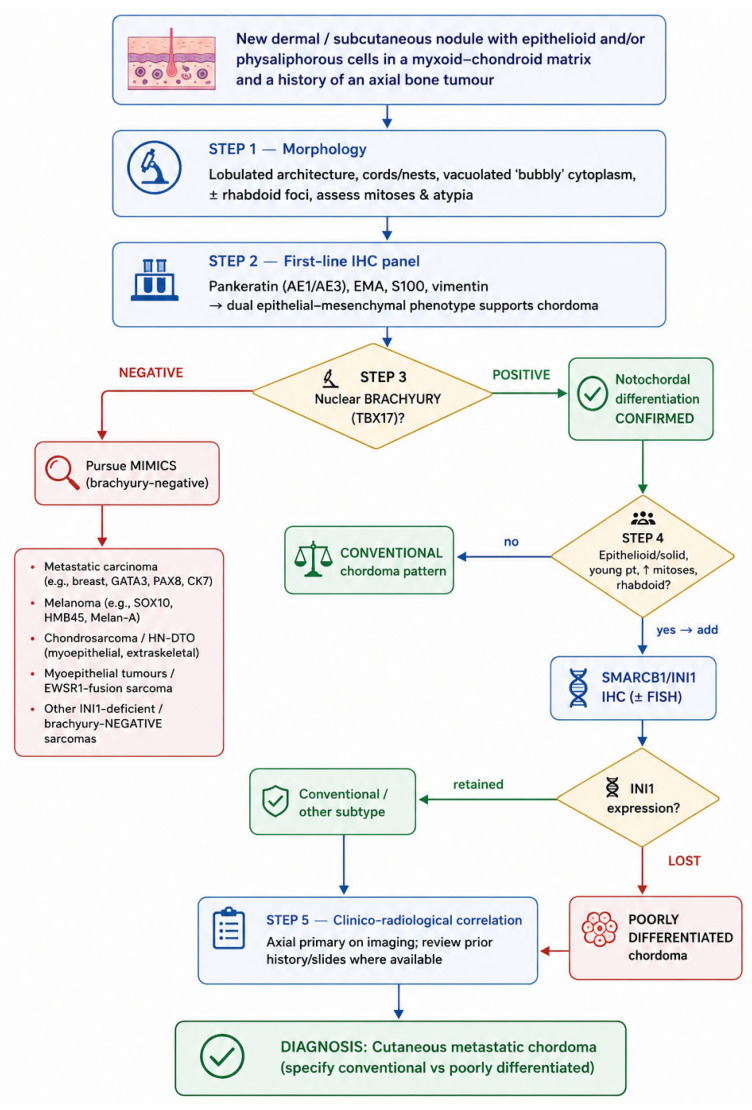
Proposed diagnostic algorithm for distant cutaneous metastasis of chordoma.

Integrating the cohort and the index case, we propose the stepwise algorithm in Figure 9. A new dermal/subcutaneous nodule with epithelioid and/or physaliphorous cells in a myxoid–chondroid matrix—especially with any history of an axial bone tumour—should trigger morphological assessment, a first-line panel (pancytokeratin, EMA, S100, vimentin) and then nuclear brachyury. Positive brachyury confirms notochordal differentiation; in epithelioid/solid tumours, in young patients, or when mitoses and rhabdoid features are present, SMARCB1/INI1 (with FISH) identifies poorly differentiated chordoma. Negative brachyury redirects the work-up along the exclusion criteria of Section 5 toward carcinoma, melanoma, chondrosarcoma, myoepithelial tumours and the INI1-deficient sarcomas. It should be emphasised that, although the morphological entry point and the demographic priors (older adult, sacrococcygeal primary, long latency) are supported by the pooled cohort, the immunohistochemical steps—above all nuclear brachyury and, in epithelioid or young presentations, SMARCB1/INI1—derive from the primary-tumour and reference literature rather than from the metastatic cases assembled here, in which these markers were applied in only 6/52 and 1/52 cases, respectively. The algorithm is therefore offered as an evidence-informed expert proposal whose molecular steps warrant prospective validation, not as a threshold derived from the cutaneous cohort.

## 6. Discussion

This review keeps distant cutaneous metastasis of chordoma in focus and shows a consistent signature across 52 pooled cases: an older adult (median 59 years) with a usually sacrococcygeal primary develops one or more firm dermal/subcutaneous nodules a median of four years later, frequently amid visceral disease. The index case is deliberately the exception that proves the rule—a 21-year-old with minimal history and an epithelioid, INI1-deficient tumour (Figure 8)—precisely the scenario in which the diagnosis is most often missed [27,28].

### 6.1. A Young, Rapidly Disseminating Subgroup and the INI1/Brachyury Imperative

The most clinically actionable result of our pooled analysis is that age sharply stratifies the timing and biology of cutaneous spread. Patients under 40 reached cutaneous metastasis a median of one year after diagnosis versus five years in older patients (*p* = 0.002 with the single congenital case excluded; *p* = 0.001 including it), were enriched for poorly differentiated, dedifferentiated or INI1-deficient tumours (*p* = 0.019), and more often presented with multiple deposits. Although the subgroup is small and these findings are hypothesis-generating, they are internally consistent and biologically coherent, and they reframe the index case not as a curiosity but as the archetype of an aggressive young-patient phenotype. Such an epithelioid, rhabdoid, INI1-deficient tumour in a young patient is far more readily labelled epithelioid sarcoma or AT/RT than chordoma, with major therapeutic consequences. Because brachyury is retained in poorly differentiated and metastatic chordoma but is absent in those mimics, early brachyury—and SMARCB1/INI1 wherever epithelioid morphology arises in a young patient or the history is incomplete—is the single most protective step [19,28]. This aligns with the recommendation to test for INI1 loss in patients under 35 years or with rapidly growing tumours [90].

### 6.2. Under-Ascertainment of Poorly Differentiated Chordoma as a Diagnostic-Era Artefact

The scarcity of confirmatory testing in the literature is not evidence that the markers are unhelpful but a function of when cases were reported: brachyury use rose from none before 2010 to roughly a third of reports after 2018 (Spearman ρ = 0.43, *p* = 0.008), while SMARCB1/INI1 has remained almost entirely unused throughout. Because poorly differentiated chordoma is defined by INI1 loss, the pre-marker literature must under-count it, which in turn biases historical subtype distributions toward “conventional/unspecified.” The practical message is that the field has corrected its brachyury habit but not yet its INI1 habit—precisely the gap that the young, epithelioid subgroup most needs closed [27,28].

### 6.3. Molecular Underpinnings and Their Diagnostic and Therapeutic Relevance

The diagnostic primacy of brachyury in this setting rests on a molecular foundation that also explains its persistence in metastatic and dedifferentiated deposits. Brachyury is not merely a lineage marker but the central driver of chordoma: germline tandem duplication of TBXT underlies familial disease, the common rs2305089 variant is carried by the great majority of patients, and somatic TBXT copy-number gain recurs in sporadic tumours, while silencing brachyury arrests chordoma cell growth [13,14,21,91]. Whole-genome studies have layered onto this otherwise genomically quiet notochordal programme recurrent alterations of the PI3K–mTOR axis, of SWI/SNF chromatin-remodelling genes and of LYST, without displacing brachyury as the unifying dependency [22]. This biology accounts for the retention of nuclear brachyury even in poorly differentiated and cutaneous deposits, and it underpins emerging brachyury-directed therapeutics—immunotherapeutic vaccines and, more recently, small-molecule transcriptional degraders—now entering clinical evaluation [92,93,94]. The poorly differentiated subtype, exemplified by the present index case and over-represented in the young, rapidly disseminating subgroup, is defined molecularly by biallelic SMARCB1 (INI1) inactivation—most often homozygous deletion—which abolishes INI1 expression and produces the epithelioid, rhabdoid phenotype that so readily mimics epithelioid sarcoma and atypical teratoid/rhabdoid tumour [27,28]. DNA-methylation profiling offers an orthogonal and increasingly accessible means of confirming notochordal identity and resolving INI1-deficient mimics when immunohistochemistry is equivocal [29]. Embedding these molecular tests in sequence—nuclear brachyury first, then SMARCB1/INI1 with confirmatory FISH and, where available, methylation class—into the work-up of any unexplained cutaneous epithelioid tumour is the single most reliable safeguard against the misdiagnoses catalogued in Section 5.

**Figure 10:**
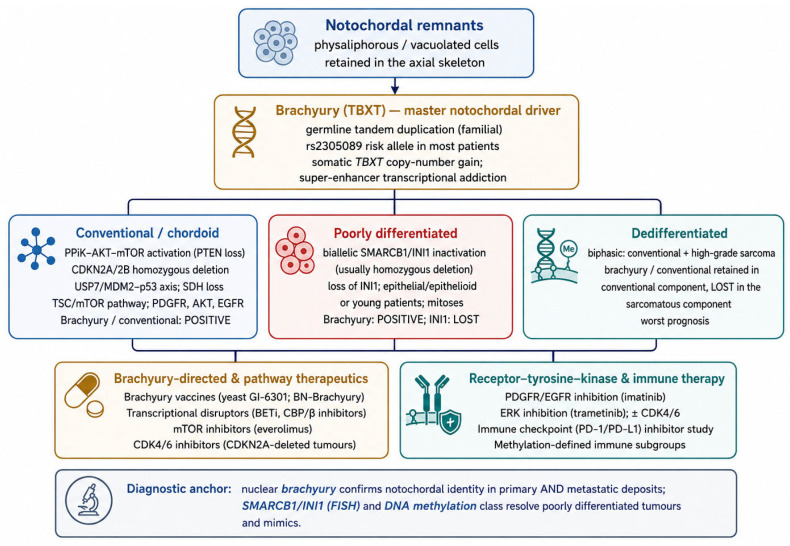
Molecular pathogenesis of chordoma and its therapeutic targets. Brachyury (TBXT) is the apex notochordal driver from which the conventional, poorly differentiated (SMARCB1/INI1-deficient) and dedifferentiated subtypes diverge; the principal co-alterations and the corresponding brachyury-directed, pathway-directed, receptor-tyrosine-kinase and immune therapies are indicated.

Read as a whole, these alterations frame chordoma as a brachyury-addicted but pathway-pleiotropic disease, and they organize an otherwise disparate therapeutic landscape (Figure 10). Because brachyury is the apex dependency, it is the most actively pursued target: yeast-based and poxvirus-vectored brachyury vaccines have reached clinical testing, and the recognition that chordoma cells are transcriptionally addicted to brachyury through a super-enhancer has motivated degrader and CDK7/9-directed strategies [91,92,95]. Downstream, PI3K–mTOR activation rationalizes mTOR inhibition (frequently combined with imatinib), recurrent CDKN2A/2B loss nominates CDK4/6 inhibition, the expressed receptor tyrosine kinases support PDGFR- and EGFR-directed agents, and a methylation-defined immune compartment underlies ongoing trials of checkpoint blockade [23,25,26,30,93,94]. Distant cutaneous dissemination, usually a marker of advanced disease, is precisely the setting in which this molecularly informed systemic menu becomes clinically relevant.

**Figure 11:**
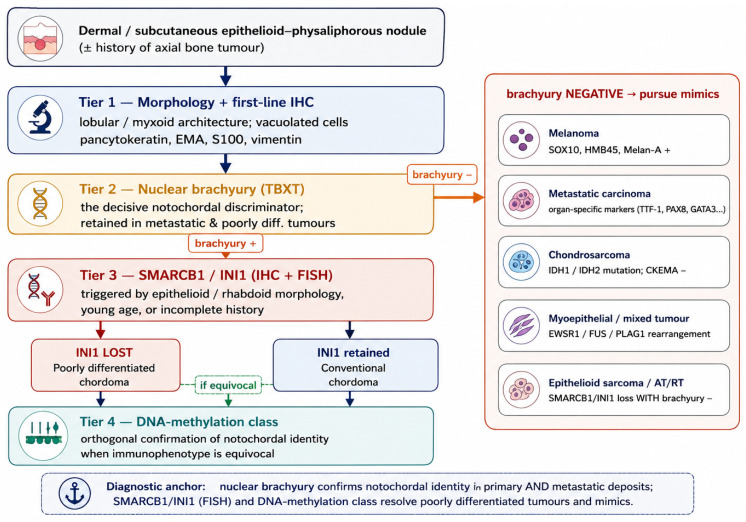
Tiered molecular work-up of a cutaneous notochordal tumour, correlated with the differential diagnosis. Nuclear brachyury separates chordoma from its mimics (each resolved by a specific molecular discriminator); SMARCB1/INI1 with FISH then identifies the poorly differentiated subtype, and DNA-methylation classification provides orthogonal confirmation when the immunophenotype is equivocal.

Three of the present findings map directly onto this biology. First, the young, rapidly and multifocally disseminating subgroup is enriched for poorly differentiated, SMARCB1/INI1-deficient tumours, tying an aggressive clinical trajectory to a defined molecular lesion and explaining why such cases are so readily mislabelled epithelioid sarcoma or atypical teratoid/rhabdoid tumour. Second, the retention of nuclear brachyury across primary and cutaneous deposits—despite extensive morphological drift—reflects the constitutive, copy-number-reinforced TBXT programme and is precisely what makes brachyury so dependable a discriminator in the skin. Third, the diagnostic-era under-ascertainment of brachyury and, especially, of SMARCB1/INI1 means that the molecular characterization of cutaneous chordoma remains incomplete; closing that gap through a tiered molecular work-up (Figure 11)—nuclear brachyury first, then SMARCB1/INI1 with confirmatory FISH and, where equivocal, DNA-methylation classification—is now both a diagnostic and a therapeutic imperative, since the same molecular axes nominate the systemic options summarized above.

### 6.4. Mechanisms, Latency and Surveillance

Cutaneous involvement arises by local extension/recurrence, surgical-track and cicatricial seeding, and haematogenous dissemination [11,12]. The long, variable latency we observed (median 4 years, up to 15) reflects the tumour’s capacity for dormancy and argues that any new cutaneous nodule in a chordoma patient—within a scar or at a distant site—warrants biopsy and a low threshold for notochordal markers, with surveillance extended well beyond conventional windows [4,5]. Because clinical and radiological surveillance may miss small dermal deposits, and because patient-related delays in seeking assessment of new skin lesions can further postpone diagnosis [96], there is growing interest in minimally invasive monitoring of skeletal and visceral malignancy—including circulating nucleic-acid and inflammatory biomarkers—that could in principle flag early dissemination, although none is yet validated in chordoma [97,98].

### 6.5. Prognosis and Management Context

Chordoma is generally chemoresistant; standard care for localised disease is en bloc resection with high-dose (proton/carbon-ion) radiotherapy, yet long-term survival is limited [32,99]. Systemic options for advanced disease are evolving and include tyrosine-kinase inhibitors and investigational brachyury-directed and immune therapies [93,94]. Reliable survival data specific to cutaneous metastasis are lacking because the evidence is confined to case reports, but distant cutaneous deposits almost always arise in the setting of disseminated disease and portend a poor outcome. For metastatic chordoma overall, disease-specific survival is on the order of 45% at five years and 25% at ten years and is strongly modified by age and subtype—dedifferentiated and poorly differentiated tumours behave far more aggressively, with dedifferentiated disease frequently fatal within a year [3,4,5]. In the pooled cohort, cutaneous metastasis typically accompanied visceral dissemination, and several historical cases recorded skin deposits only months before death; life expectancy once distant cutaneous metastasis is established is therefore best counselled as limited—commonly months to a few years—rather than the decade-scale survival occasionally seen with indolent, oligometastatic bone or lung disease [4,5]. Recognition nonetheless matters for staging, for access to the molecularly informed systemic options above, and for multidisciplinary supportive care—including management of the functional morbidity of axial and skull-base disease and attention to patient-reported quality of life—which increasingly shapes outcomes in advanced skeletal and visceral malignancy [100,101,102,103].

### 6.6. Strengths, Limitations and the Related Literature

To our knowledge this is the most focused synthesis of distant cutaneous chordoma metastasis to date, complementing the older immunohistochemical series of skin-involving chordoma [6] and the recent broader review of cutaneous involvement in metastatic bone sarcomas [8]. Its limitations are intrinsic to the source material: heterogeneous case-level reporting spanning decades, variable denominators, unavailability of brachyury/INI1 for much of the period (biasing subtype assignment), and language/publication bias. These features also bound the meta-analysis itself. Because the included reports are overwhelmingly single-patient, the random-effects models show no estimable between-report heterogeneity (I^2^ = 0%) and the pooled proportions necessarily coincide with the aggregate values; the Freeman–Tukey double-arcsine transformation is known to be unstable in this sparse-data setting, so logistic-normal estimates were preferred. The individual-participant data were extracted from published reports rather than supplied by the original investigators, so the synthesis does not meet the full PRISMA-IPD standard, and the pooled estimates and the hazard ratio for early cutaneous spread should be interpreted as a quantified consolidation of a descriptive evidence base rather than as confirmatory effect sizes. The risk-of-bias appraisal, anchored in the harmonised dataset, likewise warrants confirmation against the primary full texts. As detailed in the Methods, case-level data were frequently incomplete and were analysed on an available-case basis without imputation; denominators therefore vary across variables, and features that were merely untested—most notably brachyury and SMARCB1/INI1 in older reports—cannot be distinguished with certainty from true absence, which may bias the descriptive frequencies.

## 7. Conclusions

Distant cutaneous metastasis of chordoma is rare, late and easily misdiagnosed. It typically affects older adults with sacrococcygeal primaries after a median latency of four years but may, as here, present in a young adult as an epithelioid, INI1-deficient poorly differentiated chordoma with minimal history. Nuclear brachyury is the decisive discriminator from the full range of mimics, and SMARCB1/INI1 resolves the poorly differentiated subtype. We recommend a low threshold for these markers whenever an unusual cutaneous tumour arises in a patient with any history of an axial neoplasm [1,19,28].

## Figures and Tables

**Table 1:** Histological subtypes of chordoma and their diagnostic work-up.

Subtype	Histopathology	Ancillary Work-Up
Conventional (incl. chondroid variant)	Lobules separated by fibrous septa; cords/ribbons of physaliphorous cells in a myxoid matrix; minimal atypia; mitoses scarce. Chondroid variant has hyaline-cartilage-like matrix.	CK8/18/19, EMA, S100, vimentin, brachyury positive; CK7/CK20 negative. No recurrent diagnostic molecular alteration.
Poorly differentiated	Solid sheets/nests of epithelioid cells, eosinophilic cytoplasm with intracytoplasmic vacuoles; focal rhabdoid change; vesicular nuclei; brisk mitoses. Physaliphorous cells often absent.	Cytokeratin, EMA, brachyury positive; LOSS of SMARCB1/INI1 (FISH: homozygous SMARCB1 deletion). Younger patients; aggressive.
Dedifferentiated	Biphasic: conventional chordoma juxtaposed with high-grade sarcoma (often UPS/osteosarcoma-like). <1% of chordomas.	Brachyury/cytokeratin retained in conventional component but lost in the sarcomatous component. Worst prognosis.

**Table 2:** Pooled summary of 52 reported cases of distant cutaneous chordoma metastasis.

Variable	Category	n (Denominator)
Cases identified	Total	52 (40 reports)
	With individual patient data	43
Age, years	Median (range)	59 (2 mo–88)
	Paediatric (<18 y)	1/40
Sex	Male:Female	28:12 (2.3:1)
Primary site	Sacrococcygeal	31/42 (74%)
	Skull base/clivus/multiple	4/4/others
Subtype (when stated)	Conventional/chondroid/dediff.	6/2/2
	Not specified	33
Latency	Median (IQR), years	4.0 (1.5–6.0)
	Range	synchronous–15 y
Pattern	Solitary:multiple	21:19
Cutaneous site †	Multiple/face/scalp	17/8/5
Associated spread †	Lung/bone/liver/LN	14/9/9/5
Histology †	Physaliphorous/myxoid/vacuolated	33/31/29
Immunohistochemistry	EMA/cytokeratin/S100	24/20/19
	Brachyury performed	6
	SMARCB1/INI1 assessed	1

† Categories are not mutually exclusive. Denominators vary owing to incomplete historical reporting.

**Table 3:** Condensed evidence table of analysable cases (latency in years unless stated; +, positive; NP, not performed; NR, not reported).

Study [Ref]	Age/Sex	Primary	Latency (y)	Skin Site	Brachyury
Vaz Cunha et al., 2023 [43]	85/M	Sacrum	4	Face	NR
Jung et al., 2023 [44]	NR	Multiple	0.67	Scalp	+
Berlucchi et al., 2020 [45]	55/M	Cervical	5	Neck	NP
Ren et al., 2019 [46]	63/F	Skull base	3.5	Face	NR
Smith et al., 2018 [47]	59/M	Sacrum	10	Acral	NR
Delteil et al., 2018 [48]	84/M	Sacrum	4	Adjacent *	+
Gleghorn et al., 2017 [49]	61/M	Sacrum	8	Scalp	+
D’Amuri et al., 2017 [50]	68/F	Sacrum	10	Trunk	NR
Dahl et al., 2017 [51]	2 mo/M	Skull base	0 (synchr.)	Multiple	+
Riesco-Martínez et al., 2016 [52]	45/F	Sacrum	6	Multiple	NR
Collins et al., 2012 [53]	44/M	Sacrum	12	Trunk	NR
Svoboda et al., 2012 [54]	78/F	Sacrum	2	Multiple	NR
Agrawal et al., 2006 [55]	34/M	Sacrum	1.5	Scalp	NR
Rubin et al., 2005 [56]	70/F	Sacrum	7	Trunk	NR
Ruiz et al., 2000 [58]	62/M	Sacrum	6	Multiple	NR
Cesinaro et al., 1995 [59]	40/M	Sacrum	1.33	Face	NR
Ogi et al., 1995 [60]	22/F	Skull base	1	Leg	NR
Jones et al., 1994 [61]	67/M	Sacrum	NR	Scalp	NR
Su et al., 1993 [62]	62/F	Sacrum	5	Scalp	NR
Malone et al., 1987 [63]	65/M	Lumbar	1	Face	NR
Elliott et al., 1983 [64]	48/M	Sacrum	15	Face (orbit)	NR
Dimopoulos et al., 2023 [65]	60/M	Mobile spine	9	Face	+
Dimopoulos et al., 2023 [65]	66/M	Sacrum	6	Face	+
Vogin et al., 2016 [66]	25/F	Multiple	1.42	Multiple	NR
Vogin et al., 2016 [66]	50/M	Multiple	3.6–4.0	Multiple	NR
Vogin et al., 2016 [66]	53/M	Multiple	6.5	Arm	NR
Chambers et al., 1979 [67]	52/M	Sacrum	3	Multiple	NR
Chambers et al., 1979 [67]	28/M	Sacrum	0 (synchr.)	Multiple	NR
Chambers et al., 1979 [67]	28/M	Sacrum	0 (synchr.)	Multiple	NR
Kishimoto et al., 2012 [68]	NR	NR	3.75 (avg)	NR	NR
Chalmers et al., 1960 [69]	49/F	Sacrum	5	Multiple	NR
Kamrin et al., 1964 [70]	NR	Sacrum	NR	NR	NR
Wang et al., 1968 [71]	59/M	Sacrum	3.3	Multiple	NR
Persichetti et al., 2007 [72]	61/F	Sacrum	5	Scar	NR
Peramezza et al., 1993 [73]	61/M	Sacrum	0.5	Multiple	NR
Gattoni et al., 2005 [35]	56/M	Skull base	6	Face	NR
Benahmed et al., 2022 [75]	88/F	Sacrum	8	Leg	NR
Suárez Conde et al., 2019 [76]	60/M	Sacrum	1	Multiple	NP
Vergara et al., 2008 [77]	57/M	Sacrum	8.3	Multiple	NR
Vergara et al., 2008 [77]	63/M	Sacrum	2.2	Multiple	NR
Narang et al., 2018 [78]	61/M	Sacrum	5	Multiple	NR
Willis et al., 1930 [79]	41/F	Sacrum	4.2	Neck	NR
Yarom et al., 1970 [80]	57/M	Sacrum	3	Multiple	NR

The complete case-level dataset for all 52 cases is provided as Supplementary Table S1. Study labels and layout were regularised against Supplementary Table S1 (one row per patient). Latency in years; 0 (synchr.), synchronous presentation; avg, cohort average; +, brachyury positive; NP, not performed; NR, not reported; * lesion adjacent to the primary (local extension).

**Table 4:** Differential diagnosis of distant cutaneous metastasis of chordoma, with exclusion criteria and decisive markers.

Entity	Overlapping Feature	Exclusion Criterion (Rules Chordoma OUT/Mimic IN)	Decisive Markers
Benign notochordal cell tumour	Vacuolated/physaliphorous cells; same axial sites	Intraosseous only, NO lobular architecture, myxoid matrix, fibrous septa, mitoses, or extraosseous mass; sclerotic & non-enhancing on imaging	Brachyury+ (both); morphology/imaging decide
Chondrosarcoma (vs chondroid chordoma)	Myxoid/chondroid matrix; skull base	IDH1/IDH2 mutation present; epithelial markers and brachyury ABSENT; tends to be off-midline	S100+, CK/EMA−, brachyury−, IDH1/2 mutant
Atypical teratoid/rhabdoid tumour	Epithelioid/rhabdoid cells; SMARCB1/INI1 loss; young patients	Brachyury NEGATIVE; CNS/midline infant tumour; cytokeratin variable	INI1 loss + brachyury− indicating AT/RT; brachyury+ indicating PD chordoma
Myoepithelial tumour/mixed tumour	Myxoid stroma, epithelioid cells, keratin/S100/EMA+	Brachyury NEGATIVE; EWSR1/FUS/PLAG1 rearrangement; ductal areas in mixed tumour	Brachyury−, EWSR1/FUS/PLAG1+
Metastatic carcinoma (incl. mucinous, RCC)	Cytokeratin+, epithelioid, mucinous stroma	Organ-specific markers+; brachyury & (usually) S100 NEGATIVE; not lobulated/myxoid	TTF1/napsin, PAX8/CAIX, GATA3, CDX2; brachyury−
Melanoma (amelanotic/balloon-cell)	Vacuolated cytoplasm, S100+	Melanocytic markers+; keratin & brachyury NEGATIVE	SOX10, HMB45, Melan-A+; brachyury−
Epithelioid sarcoma	Epithelioid cells, keratin+, SMARCB1/INI1 loss	Brachyury NEGATIVE; CD34 often+; distal-extremity adult	INI1 loss + brachyury−
Chordoid meningioma	Cords in myxoid matrix, EMA+	Brachyury, cytokeratin, S100 NEGATIVE; SSTR2A/PR+; dural-based	EMA+ only; brachyury−, SSTR2A+
Myxopapillary ependymoma	Myxoid matrix, vacuoles	GFAP+; brachyury NEGATIVE; filum terminale	GFAP+, brachyury−

## Data Availability

The original contributions presented in this study are included in the article and Supplementary Materials; the complete literature-review dataset for all included cases is provided therein. Further inquiries can be directed to the corresponding authors.

## Supplementary Materials

The following supporting information can be downloaded at https://www.mdpi.com/article/10.3390/cells15151348/s1: Table S1: Complete case-level dataset of the 52 included cases of distant cutaneous metastasis of chordoma; Table S2: JBI Critical Appraisal Checklist for Case Series applied to the five multi-patient series. Each series was independently appraised by two reviewers against the ten checklist items; the single-patient case reports were appraised separately with the JBI Critical Appraisal Checklist for Case Reports (Figure 7). No report was excluded on quality grounds; Table S3: Completed PRISMA 2020 checklist for this systematic review. Completed against the PRISMA 2020 statement (Page MJ, et al. BMJ 2021;372:n71); “Location reported” indicates the corresponding section, table or figure of the manuscript.

## References

[1] WHO (2020). WHO Classification of Tumours Editorial Board: Soft Tissue and Bone Tumours.

[2] McMaster M.L., Goldstein A.M., Bromley C.M., Ishibe N., Parry D.M. (2001). Chordoma: Incidence and survival patterns in the United States, 1973–1995. Cancer Causes Control.

[3] Smoll N.R., Gautschi O.P., Radovanovic I., Schaller K., Weber D.C. (2013). Incidence and relative survival of chordomas: The standardized mortality ratio and the impact of chordoma subtype on survival. Cancer.

[4] Young V.A., Curtis K.M., Temple H.T., Eismont F.J., DeLaney T.F., Hornicek F.J. (2015). Characteristics and patterns of metastatic disease from chordoma. Sarcoma.

[5] McPherson C.M., Suki D., McCutcheon I.E., Gokaslan Z.L., Rhines L.D., Mendel E. (2006). Metastatic disease from spinal chordoma: A 10-year experience. J. Neurosurg. Spine.

[6] Gagne E.J., Su W.P. (1992). Chordoma involving the skin: An immunohistochemical study of 11 cases. J. Cutan. Pathol..

[7] Alina V.A., Maghiar O., Maghiar L., Cuc R., Pop O., Pascalau A., Boros M., Maghiar A. (2023). Cutaneous Metastasis of Rectal Adenocarcinoma: A Case Report and Literature Review. Pol. J. Pathol..

[8] Sideris N., Vakirlis E., Sotiriou E. (2026). A systematic review of cutaneous involvement in metastatic bone sarcomas: Insights from 102 reported cases. Cancers.

[9] Buhaș C.L., Mihalache G.C., Judea-Pusta C.T., Daina L.G., Muțiu G., Buhaș B.A., Popa A.R., Jurcă M.C., Nicoară N.D., Maghiar A.M. (2019). The Importance of the Histopathological Examination in Establishing the Diagnosis of Delayed Splenic Rupture. Report of a Case and Literature Review. Rom. J. Morphol. Embryol..

[10] Rakitovan M., Nicoara A., Closca R.M., Balica N.C., Stefanescu E.H., Baderca F. (2023). Leiomyoma with Uncommon Localization—Incisive Papilla and Palatal Fibromucosa: A Case Report. Medicina.

[11] Santegoeds R.G.C., Alahmari M., Postma A.A., Liebsch N.J., Weber D.C., Mammar H., Eekers D.B.P., Temel Y. (2022). Ectopic recurrence of skull base chordoma after proton therapy. Curr. Oncol..

[12] Krengli M., Poletti A., Ferrara E., Fossati P. (2016). Tumour seeding in the surgical pathway after resection of skull base chordoma. Rep. Pract. Oncol. Radiother..

[13] Vujovic S., Henderson S., Presneau N., Odell E., Jacques T.S., Tirabosco R., Boshoff C., Flanagan A.M. (2006). Brachyury, a crucial regulator of notochordal development, is a novel biomarker for chordomas. J. Pathol..

[14] Kelley M.J., Shi J., Ballew B., Hyland P.L., Li W.Q., Rotunno M., Alcorta D.A., Liebsch N.J., Mitchell J., Bass S. (2014). Characterization of T gene sequence variants and germline duplications in familial and sporadic chordoma. Hum. Genet..

[15] Wasserman J.K., Gravel D., Purgina B. (2018). Chordoma of the head and neck: A review. Head Neck Pathol..

[16] Stefanescu E.H., Balica N.C., Motoi S.B., Grigorita L., Georgescu M., Iovanescu G. (2023). High-Resolution Computed Tomography in Middle Ear Cholesteatoma: How Much Do We Need It?. Medicina.

[17] Hornick J.L. (2020). Practical Soft Tissue Pathology: A Diagnostic Approach.

[18] Oakley G.J., Fuhrer K., Seethala R.R. (2008). Brachyury, SOX-9, and podoplanin, new markers in the skull base chordoma vs chondrosarcoma differential: A tissue microarray-based comparative analysis. Mod. Pathol..

[19] Barresi V., Ieni A., Branca G., Tuccari G. (2014). Brachyury: A diagnostic marker for the differential diagnosis of chordoma and hemangioblastoma versus neoplastic histological mimickers. Dis. Markers.

[20] Sangoi A.R., Karamchandani J., Lane B., Higgins J.P., Rouse R.V., Brooks J.D., McKenney J.K. (2011). Specificity of brachyury in the distinction of chordoma from clear cell renal cell carcinoma and germ cell tumors. Mod. Pathol..

[21] Presneau N., Shalaby A., Ye H., Pillay N., Halai D., Idowu B., Tirabosco R., Whitwell D., Jacques T.S., Kindblom L.G. (2011). Role of the transcription factor T (brachyury) in the pathogenesis of sporadic chordoma: A genetic and functional-based study. J. Pathol..

[22] Tarpey P.S., Behjati S., Young M.D., Martincorena I., Alexandrov L.B., Farndon S.J., Guzzo C., Hardy C., Latimer C., Butler A.P. (2017). The driver landscape of sporadic chordoma. Nat. Commun..

[23] Hallor K.H., Staaf J., Jonsson G., Heidenblad M., von Steyern F.V., Bauer H.C.F., Ijszenga M., Hogendoorn P.C.W., Mandahl N., Szuhai K. (2008). Frequent deletion of the CDKN2A locus in chordoma: Analysis of chromosomal imbalances using array comparative genomic hybridisation. Br. J. Cancer.

[24] Le L.P., Nielsen G.P., Rosenberg A.E., Thomas D., Batten J.M., Deshpande V., Schwab J., Duan Z., Xavier R.J., Hornicek F.J. (2011). Recurrent chromosomal copy number alterations in sporadic chordomas. PLoS ONE.

[25] Tamborini E., Miselli F., Negri T., Lagonigro M.S., Staurengo S., Dagrada G.P., Stacchiotti S., Pastore E., Gronchi A., Perrone F. (2006). Molecular and biochemical analyses of platelet-derived growth factor receptor (PDGFR) B, PDGFRA, and KIT receptors in chordomas. Clin. Cancer Res..

[26] Stacchiotti S., Longhi A., Ferraresi V., Grignani G., Comandone A., Stupp R., Bertuzzi A., Tamborini E., Pilotti S., Messina A. (2012). Phase II study of imatinib in advanced chordoma. J. Clin. Oncol..

[27] Yeter H.G., Kosemehmetoglu K., Soylemezoglu F. (2019). Poorly differentiated chordoma: Review of 53 cases. APMIS.

[28] Shih A.R., Cote G.M., Chebib I., Choy E., DeLaney T., Deshpande V., Hornicek F.J., Miao R., Schwab J.H., Nielsen G.P. (2018). Clinicopathologic characteristics of poorly differentiated chordoma. Mod. Pathol..

[29] Capper D., Jones D.T.W., Sill M., Hovestadt V., Schrimpf D., Sturm D., Koelsche C., Sahm F., Chavez L., Reuss D.E. (2018). DNA methylation-based classification of central nervous system tumours. Nature.

[30] Mathios D., Ruzevick J., Jackson C.M., Xu H., Shah S., Taube J.M., Burger P.C., McCarthy E.F., Quinones-Hinojosa A., Pardoll D.M. (2015). PD-1, PD-L1, PD-L2 expression in the chordoma microenvironment. J. Neurooncol..

[31] Camarasan O.A., Camarasan A., Muresan M.M., Magheru S., Pascalau A., Pop-Crisan A., Vilceanu N., Vilceanu I., Maghiar A. (2024). CINtec PLUS: A Novel Alternative Screening Method for Detecting High-Risk Cervical Lesions in Romania. Cureus.

[32] Walcott B.P., Nahed B.V., Mohyeldin A., Coumans J.V., Kahle K.T., Ferreira M.J. (2012). Chordoma: Current concepts, management, and future directions. Lancet Oncol..

[33] Page M.J., McKenzie J.E., Bossuyt P.M., Boutron I., Hoffmann T.C., Mulrow C.D., Shamseer L., Tetzlaff J.M., Akl E.A., Brennan S.E. (2021). The PRISMA 2020 statement: An updated guideline for reporting systematic reviews. BMJ.

[34] Feier C.V.I., Gaborean V., Faur I.F., Vonica R.C., Faur A.M., Rus V.I., Dragan B.S., Muntean C. (2025). A Systematic Review of Closed-Incision Negative-Pressure Wound Therapy for Hepato-Pancreato-Biliary Surgery: Updated Evidence, Context, and Clinical Implications. J. Clin. Med..

[35] Gattoni M., Astolfi S., Tiberio R., Boldorini R., Pia F., Leigheb G. (2005). Extension cutanée d’un chordome [Cutaneous spreading of a chordoma]. Ann. Dermatol. Venereol..

[36] Stewart L.A., Clarke M., Rovers M., Riley R.D., Simmonds M., Stewart G., Tierney J.F. (2015). Preferred Reporting Items for a Systematic Review and Meta-analysis of Individual Participant Data: The PRISMA-IPD statement. JAMA.

[37] Riley R.D., Lambert P.C., Abo-Zaid G. (2010). Meta-analysis of individual participant data: Rationale, conduct, and reporting. BMJ.

[38] Schwarzer G., Chemaitelly H., Abu-Raddad L.J., Rucker G. (2019). Seriously misleading results using inverse of the Freeman-Tukey double arcsine transformation in meta-analysis of single proportions. Res. Synth. Methods.

[39] DerSimonian R., Laird N. (1986). Meta-analysis in clinical trials. Control Clin. Trials.

[40] Higgins J.P.T., Thompson S.G., Deeks J.J., Altman D.G. (2003). Measuring inconsistency in meta-analyses. BMJ.

[41] Murad M.H., Sultan S., Haffar S., Bazerbachi F. (2018). Methodological quality and synthesis of case series and case reports. BMJ Evid. Based Med..

[42] Aromataris E., Munn Z. (2020). JBI Manual for Evidence Synthesis.

[43] Vaz Cunha M., Carvalho Mendonca J., Silva Oliveira M., Lima B., Pereira O., Monteiro P. (2023). A rare case of skin metastasis from a chordoma. Dermatol. Online J..

[44] Jung D., Ko S.M., Seo J., Park E.J., Kim K.J., Kim K.H. (2023). A rare case of chordoma cutis. J. Cutan. Pathol..

[45] Berlucchi S., Nasi D., Zunarelli E., Valluzzi A., Alicandri Ciufelli M., Presutti L., Pavesi G. (2020). Cutaneous metastasis from cervical spinal chordoma: Case report and literature review. World Neurosurg..

[46] Ren H., Zhao X., Wei N., Huang H., Li W., Li H. (2019). A unique case of eyelid metastasis from chondroid chordoma. Indian J. Pathol. Microbiol..

[47] Smith Z., Girard N., Hansford B.G. (2018). Multifocal metastatic chordoma to the soft tissues of the fingertips: A case report including sonographic features and a review of the literature. Skelet. Radiol..

[48] Delteil C., Malissen N., Appay R., Magis Q., Aubert S., Bouvier C., Richard M.A., Macagno N. (2018). Chordoma cutis, an unusual clinical presentation of a rare neoplasm: Chordoma. Ann. Pathol..

[49] Gleghorn K., Goodwin B., Sanchez R. (2017). Cutaneous metastasis from sacral chordoma. Am. J. Dermatopathol..

[50] D’Amuri A., Brunelli M., Floccari F., De Caro F., Crisman G., Sanguedolce F., Filotico M. (2017). On a rare cutaneous metastasis from a sacrococcygeal chordoma. Case Rep. Pathol..

[51] Dahl N.A., Luebbert T., Loi M., Neuberger I., Handler M.H., Kleinschmidt-DeMasters B.K., Mulcahy Levy J.M. (2017). Chordoma occurs in young children with tuberous sclerosis. J. Neuropathol. Exp. Neurol..

[52] Riesco-Martinez M.C., Parrilla-Rubio L., Enguita-Valls A.B., Delgado-Marquez A.M., Ruste S.A., Lopez-Martin J.A. (2016). A unique case of distant skin metastasis from chondroid chordoma. JAAD Case Rep..

[53] Collins G.R., Essary L., Strauss J., Hino P., Cockerell C.J. (2012). Incidentally discovered distant cutaneous metastasis of sacral chordoma: A case with variation in S100 protein expression (compared to the primary tumor) and review of the literature. J. Cutan. Pathol..

[54] Svoboda R.M., Mackay D., Welsch M.J., Anderson B.E. (2012). Multiple cutaneous metastatic chordomas from the sacrum. J. Am. Acad. Dermatol..

[55] Agrawal P.P., Bahadur A.K., Mohanta P.K., Singh K., Rathi A.K. (2006). Chordoma: 6 years’ experience at a tertiary centre. Australas. Radiol..

[56] Rubin A.I., Bagel J., Niedt G. (2005). Chordoma cutis. J. Am. Acad. Dermatol..

[57] Boneschi V., Tourlaki A., Parafioriti A., Berti E., Brambati M., Brambilla L. (2003). Chordoma cutis. Eur. J. Dermatol..

[58] Ruiz H.A., Goldberg L.H., Humphreys T.R., Blacklock J.B. (2000). Cutaneous metastasis of chordoma. Dermatol. Surg..

[59] Cesinaro A.M., Maiorana A., Annessi G., Collina G. (1995). Cutaneous metastasis of chordoma. Am. J. Dermatopathol..

[60] Ogi H., Kiryu H., Hori Y., Fukui M. (1995). Cutaneous metastasis of CNS chordoma. Am. J. Dermatopathol..

[61] Jones B., Ghosh B.C., Skelton H.G. (1994). Chordoma with cutaneous metastasis. Cutis.

[62] Su W.P., Louback J.B., Gagne E.J., Scheithauer B.W. (1993). Chordoma cutis: A report of nineteen patients with cutaneous involvement of chordoma. J. Am. Acad. Dermatol..

[63] Malone T.J., Folberg R., Nerad J.A. (1987). Lumbosacral chordoma metastatic to the eyelid. Ophthalmology.

[64] Elliott E.C., McKinney S., Banks H., Fulks R.M. (1983). Aspiration cytology of metastatic chordoma. A case report. Acta Cytol..

[65] Dimopoulos Y.P., Ivan D., Prieto V.G., Aung P.P. (2023). Chordoma metastatic to skin: A report of two cases and a brief review of the literature. J. Cutan. Pathol..

[66] Vogin G., Calugaru V., Bolle S., George B., Oldrini G., Habrand J.L., Mammar H., Dendale R., Salleron J., Noel G. (2016). Investigation of ectopic recurrent skull base and cervical chordomas: The Institut Curie’s proton therapy center experience. Head Neck.

[67] Chambers P.W., Schwinn C.P. (1979). Chordoma: A clinicopathologic study of metastasis. Am. J. Clin. Pathol..

[68] Kishimoto R., Omatsu T., Hasegawa A., Imai R., Kandatsu S., Kamada T. (2012). Imaging characteristics of metastatic chordoma. Jpn. J. Radiol..

[69] Chalmers J., Coulson W.F. (1960). A metastasising chordoma. J. Bone Jt. Surg. Br..

[70] Kamrin R.P., Potanos J.N., Pool J.L. (1964). An evaluation of the diagnosis and treatment of chordoma. J. Neurol. Neurosurg. Psychiatry.

[71] Wang C.C., James A.E. (1968). Chordoma: Brief review of the literature and report of a case with widespread metastases. Cancer.

[72] Persichetti G., Walling H.W., Rosen L., Cronin T., Ceilley R.I. (2007). Chordoma cutis. Dermatol. Surg..

[73] Peramezza C., Cellini A., Berardi P., Benvenuti S., Offidani A. (1993). Chordoma with multiple skin metastases. Dermatology.

[74] Unnikrishnan P., Nair S.G., Thambi S.M., Benson R., Mony R.P. (2019). Cutaneous metastasis from sacral chordoma: A case report. Iran. J. Blood Cancer.

[75] Benahmed J., Gil H., Charles J., Trabelsi S. (2022). Dermatoscopic findings of cutaneous chordomas mimicking cutaneous metastasis of melanoma. JAAD Case Rep..

[76] Suarez Conde M.F., Vallone M.G., Gonzalez V.M., Vigovich F.A., Casas J.G., Cermignan L., Bas C.A., Larralde M. (2019). Metastatic chordoma. JAAD Case Rep..

[77] Vergara G., Belinchon B., Valcarcel F., Veiras M., Zapata I., de la Torre A. (2008). Metastatic disease from chordoma. Clin. Transl. Oncol..

[78] Narang N.C., Narang S., Diwaker P. (2018). Multiple cutaneous metastasis of sacral chordoma, mimicking neurofibromas clinically. Ann. Pathol. Lab. Med..

[79] Willis R.A. (1930). Sacral chordoma with widespread metastases. J. Pathol. Bacteriol..

[80] Yarom R., Horn Y. (1970). Sacrococcygeal chordoma with unusual metastases. Cancer.

[81] Wiacek M.P., Kaczmarek K., Sulewski A., Kubaszewski L., Kaczmarczyk J. (2014). Unusual location of chordoma metastasis. Pol. Orthop. Traumatol..

[82] Iovănescu G., Bîrsăşteanu F., Borugă V.M., Apostol A., Ştefănescu E.H., Budu V.A., Baderca F., Trifu S.C., Mogoantă C.A., Bonţe D.C. (2020). Clinical, Ultrasound and Histopathological Correlation of Clinically N0 Neck Nodes in Patients with Cancers of the Pharynx and Larynx. Rom. J. Morphol. Embryol..

[83] Yamaguchi T., Iwata J., Sugihara S., McCarthy E.F., Karita M., Murakami H., Kawahara N., Tsuchiya H., Tomita K. (2008). Distinguishing benign notochordal cell tumors from vertebral chordoma. Skelet. Radiol..

[84] Deshpande V., Nielsen G.P., Rosenthal D.I., Rosenberg A.E. (2007). Intraosseous benign notochord cell tumors (BNCT): Further evidence supporting a relationship to chordoma. Am. J. Surg. Pathol..

[85] Yamaguchi T., Suzuki S., Ishiiwa H., Ueda Y. (2004). Intraosseous benign notochordal cell tumours: Overlooked precursors of classic chordomas?. Histopathology.

[86] Amary M.F., Bacsi K., Maggiani F., Damato S., Halai D., Berisha F., Pollock R., O’Donnell P., Grigoriadis A., Diss T. (2011). IDH1 and IDH2 mutations are frequent events in central chondrosarcoma and central and periosteal chondromas but not in other mesenchymal tumours. J. Pathol..

[87] Varachev V., Shekhtman A., Guskov D., Rogozhin D., Zasedatelev A., Nasedkina T. (2024). Diagnostics of IDH1/2 mutations in intracranial chondroid tumors: Comparison of molecular genetic methods and immunohistochemistry. Diagnostics.

[88] Calonje E., Brenn T., Lazar A.J., Billings S.D. (2020). McKee’s Pathology of the Skin.

[89] Patterson J.W. (2021). Weedon’s Skin Pathology.

[90] Chordoma Foundation Chordoma in Children and Young Adults; Medical Advisory Board Guidance Recommending SMARCB1/INI1 Testing in Patients Under 35 Years or with Rapidly Growing Tumors. https://www.chordomafoundation.org.

[91] Pillay N., Plagnol V., Tarpey P.S., Lobo S.B., Presneau N., Szuhai K., Halai D., Berisha F., Cannon S.R., Mead S. (2012). A common single-nucleotide variant in T is strongly associated with chordoma. Nat. Genet..

[92] Sharifnia T., Wawer M.J., Chen T., Huang Q.-Y., A Weir B., Sizemore A., A Lawlor M., Goodale A., Cowley G.S., Vazquez F. (2019). Small-molecule targeting of brachyury transcription factor addiction in chordoma. Nat. Med..

[93] Meng T., Jin J., Jiang C., Huang R., Yin H., Song D., Cheng L. (2019). Molecular targeted therapy in the treatment of chordoma: A systematic review. Front. Oncol..

[94] Golding R., Abuqubo R., Pansa C.J., Bhatta M., Shankar V., Mani K., Kleinbart E., Gelfand Y., Murthy S., De la Garza Ramos R. (2024). Immunologic and targeted molecular therapies for chordomas: A narrative review. J. Clin. Med..

[95] Heery C.R., Singh B.H., Rauckhorst M., Marte J.L., Donahue R.N., Grenga I., Rodell T.C., Dahut W., Arlen P.M., Madan R.A. (2015). Phase I trial of a yeast-based therapeutic cancer vaccine (GI-6301) targeting the transcription factor brachyury. Cancer Immunol. Res..

[96] Cote A., Negrut R.L., Feder B., Antal I.A., Horgos M.S., Tomescu E., Maghiar A.M. (2025). Barriers to Seeking Medical Care for Hemorrhoidal Symptoms: A Cross-Sectional Observational Study. J. Clin. Med..

[97] Negrut R.L., Cote A., Feder B., Bodog F.D., Maghiar A.M. (2025). Comparative Prognostic Role of PLR and NLR in Colon Cancer: A Retrospective Analysis of Preoperative Inflammatory Markers. Medicina.

[98] Stefanescu H., Muntean D., Pilut C., Diaconu M., Popescu R., Hutanu D., Moise M.L., Diana L., Nitu R., Cherecheanu A.P. (2018). Using Blood and Plasma MicroRNAs as a Non-Invasive Biomarker in Patients with Colorectal Cancer. Clin. Lab..

[99] Stacchiotti S., Sommer J., Chordoma Global Consensus Group (2015). Building a global consensus approach to chordoma: A position paper from the medical and patient community. Lancet Oncol..

[100] Debucean D., Mihaiu J., Maghiar A.M., Marcu F., Marcu O.A. (2023). A Multidisciplinary Approach to Swallowing Rehabilitation in Patients with Forward Head Posture. Medicina.

[101] Rat L.A., Ghitea T.C., Maghiar A.M. (2025). Effectiveness of a Self-Esteem Enhancement Intervention Integrated into Standard CBT Protocol for Improving Quality of Life in Patients with Colorectal Cancer. Eur. J. Investig. Health Psychol. Educ..

[102] Rat L.A., Ghitea T.C., Maghiar A.M. (2025). Psychological Distress and Quality of Life in Patients with Colon Cancer: Predictors, Moderating Effects, and Longitudinal Impact. Healthcare.

[103] Tarta C., Marian M., Capitanio M., Faur F.I., Duta C., Diaconescu R., Oprescu-Macovei A.M., Totolici B., Dobrescu A. (2022). The Challenges of Colorectal Cancer Surgery during the COVID-19 Pandemic in Romania: A Three-Year Retrospective Study. Int. J. Environ. Res. Public Health.

