# Supplementary material for: Distant Cutaneous Metastasis of Chordoma: A Systematic Review and Individual-Participant-Data Meta-Analysis of 52 Cases, with an Illustrative Poorly Differentiated Index Case
Source: Cells. 2026 Jul 27;15(15):1348. doi: 10.3390/cells15151348 (PMC13465574; doi:10.3390/cells15151348)
Supplement: Supplementary file 1 [file cells-15-01348-s001.zip › cells-4434988-supplementary.pdf]

Table S1. Complete case-level dataset of the 52 included cases of distant cutaneous metastasis of chordoma

Systematic review and individual-participant-data meta-analysis (40 reports, 52 cases). Cells are reported as recorded in the source publications; NR = not reported / not specified.

Case	Reference	DOI	Age (yr)	Sex	Primary chordoma site	Histological subtype	Latency to cutaneous metastasis	Cutaneous metastasis site	Other (visceral) metastases	Clinical presentation	Clinical manifestation	Lesion pattern	Lesion evolution	Morphology	Positive IHC	Negative IHC	Histochemistry	Molecular / ancillary tests
1	Vaz Cunha et al., 2023	10.5070/D329461862	85	Male	Sacrum / sacrococcygeal	NR	Approximately 4 years from the initial diagnosis of sacral chordoma to cutaneous metastasis. The primary chordoma was diagnosed in May 2016, followed by lung metastases and later skin metastasis.	Face - Left nasal pyramid base / left nasal base	Lung; later multiple nodules on the left thigh and right cervical lymphadenopathy	Nodule – erythematous; 20 × 9 mm erythematous and pseudocystic nodule	Not specified for the nasal lesion; later lesions on the left thigh were painful	Solitary lesion	1-month history of the nasal lesion	Spindle cell component; lobulated/encapsulated groups of tumor cells; morphology compatible with previously diagnosed chordoma. The article's general description of chordoma also mentions cords/clusters, mucinous/myxoid matrix, vesicular nuclei, and vacuolated "soap bubble-like" cytoplasm, but these were not all specifically confirmed for this patient's cutaneous metastasis.	Cytokeratins AE1/AE3; cytokeratins 8/18; EMA; vimentin; CD10; p63 (focal); Ki67 extensive/high staining	Cytokeratin 5; cytokeratin 7; cytokeratin 20; MOC31; p40; CD117; S100 protein; MelanA; PSA; HepPar1; TTF1	NR	NR
2	Jung et al., 2023	10.1111/cup.14525	NR	NR	Multiple primary sites	NR	8 months = ~0.67 years	Scalp	Skull involvement mentioned; vertebral column and skull chordoma diagnosed 8 months prior. Other visceral metastases not reported.	Other — translucent nodule, Solitary translucent nodule on the scalp	NR	Solitary translucent nodule on the scalp	NR	Cords / nests of tumor cells; plasmacytoid cells; myxoid stroma; physaliphorous cells absent	Brachyury; EMA	Cytokeratin AE1/AE3	NR	NR
3	Berlucchi et al., 2020	10.1016/j.wneu.2020.02.018	55	Male	Cervical spine	Conventional	5	Neck	Base of neck, approximately 1 cm from the first anterior surgical incision	No other metastases reported	Other – firm dome-shaped nodule	1-cm firm, dome-shaped nodule on the base of the neck, reported as painful and located approximately 1 cm from the first anterior surgical incision	Painful	Solitary lesion	Appeared 5 years after first surgery and 6 months after proton therapy	Physaliphorous cells; myxoid stroma; lobulated architecture; cords/nests of tumor cells; epithelioid cells; vacuolated cytoplasm; ovoid cells	pancytokeratin; EMA; vimentin; S100	Neuroepithelial markers; melanocytic markers; smooth muscle markers; lymphocytic markers
4	Ren et al., 2019	10.4103/IJPM.IJPM_387_18	63	Female	Skull base (clivus)	Conventional (chondroid variant)	~3.5	Face; Lower eyelid of right eye / subcutaneous tissue of right eyelid	Not reported; locoregional skull base recurrence reported	Nodule – erythematous; Firm, nontender erythematous nodule, 1.5 × 1 × 0.7 cm, under the lower lid skin of the right eye	Asymptomatic; nontender	Solitary lesion	Not specified for eyelid lesion; locoregional recurrence at 27 months	Lobulated architecture; chondroid stroma/matrix; physaliphorous-like "liquid drop" cells; small round/cubic cells; eosinophilic cytoplasm; mixed morphology	AE1/AE3; EMA; S100; Ki67 ~10% / Ki67 positive	CD68; CD117	NR	NR
5	Smith et al., 2018	10.1007/s00256-017-2785-4	59	Male	Sacrum / sacrococcygeal	NR	10	Distal extremity (hand/foot)	Multiple left fingertips / distal volar soft tissues of the left thumb, index, ring, and small fingers	Lung / pulmonary metastases	Other – swelling and discoloration	Multifocal soft tissue swelling with erythematous/violaceous discoloration of multiple left fingertips, most pronounced at the ring and small fingers; cyst-like masses; ultrasound showed solid circumscribed macrolobulated hypoechoic masses with hyperechoic vascular septations	Mildly tender; not painful at rest; inflammatory-like	Multiple lesions	Symptoms began 3 months before presentation and worsened significantly over the previous week; primary sacral chordoma diagnosed 10 years earlier	Physaliphorous cells; vacuolated cytoplasm; lobulated architecture; fibrous septations; high cellularity	NR	NR
6	Delteil et al., 2018	10.1016/j.annpat.2018.01.004	84	Male	Sacrum / sacrococcygeal	NR	4	Adjacent to primary tumor	Upper gluteal cleft / posterior sacral skin	No distant metastases reported; staging work-up was negative	Ulcerated lesion	Widely ulcerated lesion of the upper gluteal cleft; MRI showed a 14 cm sacral mass involving the entire sacrum with extension to posterior muscles and skin	NR	Solitary lesion	Slow progression over 4 years; primary sacral chordoma measured 8 cm initially and later progressed to a 14 cm mass extending to the skin	Physaliphorous cells; myxoid stroma; lobulated architecture; cords/nests of tumor cells; epithelioid cells; vacuolated cytoplasm; hyperchromatic nuclei; hemorrhagic changes	AE1/AE3; EMA; S100; brachyury	NR
7	Gleghorn et al., 2017	10.1097/DAD.0000000000000757	61	Male	Sacrum / sacrococcygeal	NR	8	Scalp	Left occipital scalp	No other metastases reported; further imaging was conducted to rule out other sites of metastasis	Nodule – rose/pink	1-cm rubbery, pink, shiny dome-shaped nodule on the left occipital scalp, present for 6 weeks	NR	Solitary lesion	6-week history; developed 8 years after wide local excision of primary sacral chordoma	Physaliphorous cells; myxoid stroma; lobulated architecture; ovoid cells; vacuolated "soap-bubble" cytoplasm; hyperchromatic displaced nuclei	S100; pancytokeratin AE1/AE3; brachyury	SMA; p63; CK7
8	D'Amuri et al., 2017	10.1155/2017/5281239	68	Female	Sacrum / sacrococcygeal	Conventional	10	Trunk	Right dorsal region / upper back near scapular region	NR	Other – painless subcutaneous mass	Painless subcutaneous mass in the right dorsal region, upper back near scapular region; solid ovoid subcutaneous lesion with well-defined margins, 4 cm diameter	Asymptomatic; painless	Solitary lesion	Few months; cutaneous metastasis appeared 10 years after primary sacral chordoma excision	Physaliphorous cells; myxoid stroma; lobulated architecture; cords/nests of tumor cells; vacuolated cytoplasm; mild nuclear atypia/pleomorphism; mitotic activity – low; pleomorphic cells; PAS-positive vacuolated cytoplasm	S100; cytokeratin AE1/AE3; EMA; vimentin	CEA; smooth muscle actin
9	Dahl et al., 2017	10.1093/jnen/nlx032	2 months	Male	Skull base (clivus)	Conventional	0 / synchronous at presentation	Multiple cutaneous sites	Scalp, trunk, lower extremities, and chest wall skin/subcutaneous nodule	Lungs; liver; spleen; paraspinal muscles; subcutaneous tissues	Other – firm blue-colored skin nodules	Firm blue-colored skin nodules on scalp, trunk, and lower extremities, present at birth and increased in size and number over time; chest wall skin nodule biopsied	NR	Multiple lesions	Present at birth; grew in size and number over time	Physaliphorous cells; myxoid/mucinous stroma; lobulated architecture; cords/nests of tumor cells; vacuolated cytoplasm; no atypical or dedifferentiated features; mitotic activity – low/absent	EMA; S100; AE1/AE3; brachyury; INI1 retained	NR
10	Riesco-Martínez et al., 2016	10.1016/j.jdcr.2015.11.014	45	Female	Sacrum / sacrococcygeal	Conventional (chondroid variant)	~6	Multiple cutaneous sites	Left cheek; later chin, frontal area, and back	Liver; bone; lung; soft tissue metastasis at L4 level	Nodule – erythematous	Firm, nontender, erythematous nodule measuring 2 × 2 cm on the left cheek; later two new skin lesions on the chin and frontal area and two new lesions on the back	Nontender	Multiple lesions	Progressive disease; initial cheek lesion appeared approximately 6 years after primary diagnosis; additional skin lesions appeared 7 months later	Physaliphorous cells; myxoid/chondral stroma; chondroid stroma/matrix; lobulated architecture; nests of tumor cells; vacuolated cytoplasm; eosinophilic cytoplasm; no mitosis reported in primary tumor	pancytokeratin; vimentin; S100; EMA (focal in primary tumor; positive in skin metastasis)	CEA; HMB-45
11	Collins et al., 2012	10.1111/j.1600-0560.2012.01895.x	44	Male	Sacrum / sacrococcygeal	Conventional	12	Trunk	Right upper back	Lung; bone; soft tissue; right shoulder soft tissue; posterior right chest wall; bilateral pleura and lungs; left abdominal wall; inferior pelvis	Nodule – rose/pink	Solitary 4-mm pink, shiny, slightly raised papule on the right upper back, away from the midline and unrelated to any prior scar	Asymptomatic	Solitary lesion	Incidentally discovered 12 years after primary sacral chordoma diagnosis	Physaliphorous cells; myxoid/mucoid stroma; lobulated architecture; cords/nests of tumor cells; sheets of tumor cells; vacuolated cytoplasm; epithelioid/polygonal cells; mild to moderate nuclear atypia/pleomorphism	pancytokeratin; vimentin; EMA (patchy, weak)	S100; CEA; pan-melanoma cocktail; SMA
12	Svoboda et al., 2012	10.1016/j.jaad.2011.07.037	78	Female	Sacrum / sacrococcygeal	NR	2	Multiple cutaneous sites	Scalp; left forearm	Bone metastases: L2 vertebra, T7 vertebra, right scapula, left 10th rib; sacral recurrence	Nodule – skin-colored	Multiple rubbery, flesh-colored nodular lesions of the scalp measuring 0.9 × 0.7 × 0.5 cm, 0.6 × 0.5 × 0.5 cm, and 0.5 × 0.4 × 0.4 cm; one rubbery gray nodule on the left forearm measuring 0.8 × 0.7 × 0.6 cm	NR	Multiple lesions	2 years after wide local resection of sacral chordoma	Physaliphorous cells; myxoid stroma; chondroid stroma/matrix; lobulated architecture; cords/nests of tumor cells; epithelioid cells; vacuolated "soap-bubble" cytoplasm; cuboidal epithelioid cells; fibrous bands; hyalinized myxoid chondroid matrix	NR	NR
13	Agrawal et al., 2006	10.1111/j.1440-1673.2006.01562.x	34	Male	Sacrum / sacrococcygeal	NR	1.5	Scalp	Occipital scalp / subcutaneous tissue over scalp	Lung / multiple pulmonary metastases; local recurrence	NR	Subcutaneous scalp metastasis in the occipital region, associated with multiple lung metastases and local recurrence	NR	Solitary lesion	18 months after local treatment	NR	NR	NR
14	Rubin et al., 2005	10.1016/j.jaad.2004.07.055	70	Female	Sacrum / sacrococcygeal	NR	7	Trunk	Left side of the back, 20 cm lateral to the spinal column	Liver; spleen; locally recurrent disease	Other – firm nontender nodule	Firm, nontender, 1.2 × 1.0 cm nodule on the left side of the back, 20 cm lateral to the spinal column	Nontender	Solitary lesion	Developed 7 years after initial diagnosis of sacral chordoma	Physaliphorous cells; myxoid/mucinous stroma; cords/nests of tumor cells; moderate nuclear atypia/pleomorphism; mitotic activity – low/occasional	S100; cytokeratin; EMA	NR
15	Boneschi et al., 2003	NR	NR	NR	NR	NR	NR	NR	NR	NR	NR	NR	NR	NR	NR	NR	NR	NR
16	Ruiz et al., 2000	10.1046/j.1524-4725.2000.09216.x	62	Male	Sacrum / sacrococcygeal	NR	6	Multiple cutaneous sites	Left scapular/back region; left side of nasal columella	Lung; soft tissue of posterior chest wall; right triceps tendon	Nodule – erythematous	2.5 cm round erythematous asymptomatic nodule at the medial and inferior margin of the left scapula; later erythematous nodule on the left side of the nasal columella	Asymptomatic	Multiple lesions	First skin metastasis appeared 6 years after primary sacral chordoma diagnosis; additional nasal lesion developed approximately 4 years later	Physaliphorous cells; myxoid/mucoid stroma; chondroid matrix; lobulated architecture; cords/nests of tumor cells; vacuolated cytoplasm; pleomorphic cells; high nuclear atypia/pleomorphism; increased vascularity	EMA; broad-spectrum cytokeratin; vimentin	PAS; S100 negative in skin metastases
17	Cesinaro et al., 1995	10.1097/00000372-199512000-00014	40	Male	Sacrum / sacrococcygeal	NR	1.33	Face	Right side of the nose	NR	Other – small hard cutaneous nodule	Small hard cutaneous nodule on the right side of the nose, slowly growing over 2 months, approximately 5 mm in diameter, with slightly erythematous overlying skin and no ulceration	NR	Solitary lesion	Slow growth over 2 months; developed 16 months after radical excision of sacrococcygeal chordoma	Physaliphorous cells; myxoid/mucinous stroma; cords/nests of tumor cells; vacuolated cytoplasm; hyperchromatic nuclei; eosinophilic cytoplasm; well-circumscribed dermal nodule	CAM 5.2 / low molecular weight keratins; vimentin; S100	NR
18	Ogi et al., 1995	10.1097/00000372-199512000-00013	22	Female	Skull base (clivus)	NR	1	Lower extremity	Medial aspect of the left thigh	Lung; lymph nodes; pelvic cavity; cervical vertebrae	Other – tender subcutaneous nodule	Elastic, hard, tender, fairly movable subcutaneous nodule, approximately 4 cm in diameter, in the medial aspect of the left thigh	Tender	Solitary lesion	Appeared 1 year after initial cranial operation; later recurrent mass developed at the same subcutaneous site	Physaliphorous cells; myxoid stroma; cords/nests of tumor cells; stellate cells; vacuolated cytoplasm; lobulated architecture; small polygonal, round, and stellate cells; eosinophilic cytoplasm; slightly basophilic cytoplasm	S100; cytokeratin	NR
19	Jones et al., 1994	NR	67	Male	Sacrum / sacrococcygeal	NR	NR	Scalp	Scalp	NR	NR	Scalp metastasis from chordoma	NR	Solitary lesion	NR	NR	NR	NR
20	Su et al., 1993	10.1016/0190-9622(93)70153-k	62	Female	Sacrum / sacrococcygeal	NR	5	Scalp	Left frontoparietal scalp	NR	NR	Nodular lesion in the left frontoparietal scalp, reported as distant cutaneous metastasis from sacrococcygeal chordoma	NR	Solitary lesion	Occurred 5 years after diagnosis and surgical removal of the primary sacrococcygeal chordoma	Physaliphorous cells; myxoid/mucinous stroma; cords/nests or strands/masses of tumor cells; vacuolated cytoplasm; polygonal eosinophilic cells; signet-ring-like cells occasionally described	Cytokeratin; EMA (occasional cells); keratin; vimentin; S100 in 73% of available cases	CEA
21	Malone et al., 1987	10.1016/s0161-6420(87)33339-1	65	Male	Lumbar spine	NR	~1	Face	Left lower eyelid	No other metastases reported; metastatic survey showed no other foci outside the pelvis	Other – eyelid cyst	10 × 5 × 5 mm cystic lesion of the left lower eyelid, enlarging over 4 months, translucent and fixed medially to the tarsus	NR	Solitary lesion	Enlarged over 4 months; appeared approximately 1 year after radiation therapy for lumbosacral chordoma	Physaliphorous cells; cords of tumor cells; vacuolated/bubbly cytoplasm; cystic architecture; acid mucopolysaccharide-positive tumor matrix	NR	NR
22	Elliott et al., 1983	10.1016/S0002-9394(00)00775-3	48	Male	Sacrum / sacrococcygeal	NR	15	Face	Left orbit	No other metastases reported; isolated orbital metastasis from recurrent sacrococcygeal chordoma	Other – orbital swelling/proptosis	Left orbital tumor presenting with left eye swelling, proptosis, deteriorating vision, and inability to walk; imaging showed metastatic tumor between the globe and orbital roof	NR	Solitary lesion	Developed 15 years after excision and radiotherapy for sacrococcygeal chordoma	Physaliphorous cells; myxoid stroma; lobulated architecture; vacuolated cytoplasm; round to oval cells; vesicular nuclei; thick fibrillar stromal bands; macronucleoli	NR	NR
23	Dimopoulos et al., 2023	10.1111/cup.14348	60	Male	Mobile spine (unspecified)	Conventional; features concerning for dedifferentiation in primary tumor	9	Face	Left lower jaw	No other metastases reported; skin was the only metastatic site	Other – cutaneous lesion	0.9-cm lesion on the skin of the left lower jaw; clinically considered pigmented basal cell carcinoma versus inflammatory nodule	NR	Solitary lesion	Developed 9 years after initial diagnosis of thoracic/lumbar chordoma	Lobulated architecture; epithelioid cells; physaliphorous cells; vacuolated cytoplasm; myxoid stroma; dermal and subcutaneous involvement	brachyury	NR
24	Dimopoulos et al., 2023	10.1111/cup.14348	66	Male	Sacrum / sacrococcygeal	Conventional	6	Face	Right side of chin	Liver; lung; local recurrence at L5 region	Other – firm subcutaneous nodule	3-cm irregular, firm subcutaneous nodule on the right chin; clinically considered metastatic chordoma versus cyst	NR	Solitary lesion	Developed 6 years after radical resection of sacral chordoma	Lobulated architecture; epithelioid cells; physaliphorous cells; vacuolated cytoplasm; myxoid stroma; dermal involvement; infarct-type necrosis in primary tumor	brachyury	NR
25	Vogin et al., 2016	10.1002/hed.24199	25	Female	Multiple primary sites	NR	1.42	Multiple cutaneous sites	Scalp; neck / left cervical subcutaneous tissue	Intradural dissemination; prevertebral relapse; intramuscular location	NR	Subcutaneous scalp and cervical relapses after cervico-clival chordoma; fine-needle biopsy of the subcutaneous cervical lesion identified chordoma cells	NR	Multiple lesions	17 months after primary surgery	NR	NR	NR
26	Vogin et al., 2016	10.1002/hed.24199	50	Male	Multiple primary sites	NR	3.6–4.0	Multiple cutaneous sites	Scalp; retroauricular region	No other metastases reported; isolated subcutaneous recurrence	NR	Isolated subcutaneous recurrence involving scalp and retroauricular region after skull base/cervical chordoma	NR	Multiple lesions	Reported as 43 months in Table 1 and 48 months in text after primary treatment	NR	NR	NR
27	Vogin et al., 2016	10.1002/hed.24199	53	Male	Multiple primary sites	NR	6.5	Upper extremity	Right forearm	No other metastases reported; isolated subcutaneous recurrence	NR	Isolated subcutaneous metastasis in the right forearm after clival/cervical chordoma	NR	Solitary lesion	78 months after surgery and radiation of the primary chordoma	NR	NR	NR
28	Chambers et al., 1979	10.1093/ajcp/72.5.765	52	Male	Sacrum / sacrococcygeal	NR	3	Multiple cutaneous sites	Scapular area; multiple dermal lesions, exact full distribution not reported	NR	Other – multiple dermal nodules	Multiple dermal metastases; at least four skin lesions; initially diagnosed as mixed tumors of the skin	NR	Multiple lesions	First metastasis reported 3 years after diagnosis; additional metastatic events at 4 and 5 years	Physaliphorous cells; myxoid/mucinous stroma; lobulated architecture; cords/nests of tumor cells; vacuolated cytoplasm	NR	NR
29	Chambers et al., 1979	10.1093/ajcp/72.5.765	28	Male	Sacrum / sacrococcygeal	Dedifferentiated component / fibrosarcomatous focus described	0	Multiple cutaneous sites	Multiple dermal lesions, exact sites not fully reported	NR	Other – multiple dermal nodules	Multiple dermal metastases; at least four skin lesions; initially diagnosed as mixed tumors of the skin	NR	Multiple lesions	Dermal metastases present at or before diagnosis of the primary lesion	Physaliphorous cells; myxoid/mucinous stroma; lobulated architecture; cords/nests of tumor cells; vacuolated cytoplasm; dedifferentiated component; spindle cell/fibrosarcomatous focus	NR	NR
30	Chambers et al., 1979	10.1093/ajcp/72.5.765	28	Male	Sacrum / sacrococcygeal	Dedifferentiated component / fibrosarcomatous focus described	0	Multiple cutaneous sites	Multiple dermal lesions, exact sites not fully reported	NR	Other – multiple dermal nodules	Multiple dermal metastases; at least four skin lesions; initially diagnosed as mixed tumors of the skin	NR	Multiple lesions	Dermal metastases present at or before diagnosis of the primary lesion	Physaliphorous cells; myxoid/mucinous stroma; lobulated architecture; cords/nests of tumor cells; vacuolated cytoplasm; dedifferentiated component; spindle cell/fibrosarcomatous focus	NR	NR
31	Kishimoto et al., 2012	10.1007/s11604-012-0086-3	NR	NR	NR	NR	Average 3.75 years; range 0–50 years for first metastasis detection in metastatic cohort	NR	Skin metastases reported in 7 patients, exact cutaneous sites not reported	Bone; lung; liver; lymph node; muscle; pleura; cerebellum; cardiac muscle; pericardium; adrenal gland	NR	Imaging cohort of metastatic chordoma; skin listed among metastatic sites in 7 patients, but individual cutaneous cases not described	NR	Indeterminate	Metastases detected from 0 to 600 months after diagnosis; average 45 months	NR	NR	NR
32	Kishimoto et al., 2012	10.1007/s11604-012-0086-3	NR	NR	NR	NR	NR	NR	NR	NR	NR	NR	NR	NR	NR	NR	NR	NR
33	Kishimoto et al., 2012	10.1007/s11604-012-0086-3	NR	NR	NR	NR	NR	NR	NR	NR	NR	NR	NR	NR	NR	NR	NR	NR
34	Kishimoto et al., 2012	10.1007/s11604-012-0086-3	NR	NR	NR	NR	NR	NR	NR	NR	NR	NR	NR	NR	NR	NR	NR	NR
35	Kishimoto et al., 2012	10.1007/s11604-012-0086-3	NR	NR	NR	NR	NR	NR	NR	NR	NR	NR	NR	NR	NR	NR	NR	NR
36	Kishimoto et al., 2012	10.1007/s11604-012-0086-3	NR	NR	NR	NR	NR	NR	NR	NR	NR	NR	NR	NR	NR	NR	NR	NR
37	Kishimoto et al., 2012	10.1007/s11604-012-0086-3	NR	NR	NR	NR	NR	NR	NR	NR	NR	NR	NR	NR	NR	NR	NR	NR
38	Chalmers et al., 1960	10.1302/0301-620X.42B3.556	49	Female	Sacrum / sacrococcygeal	NR	5	Multiple cutaneous sites	Left arm subcutaneous tissue; scar over the sacrum	Skeletal muscle metastases: right triceps, second right triceps lesion, brachialis	Other – small subcutaneous metastasis	Small subcutaneous metastasis in the left arm and small metastasis in the scar over the sacrum, occurring after multiple skeletal muscle metastases	NR	Multiple lesions	First metastasis appeared 5 years after primary sacral chordoma excision; subcutaneous and scar metastases appeared within the following year	Physaliphorous cells; myxoid/mucoid stroma; lobulated architecture; polyhedral cells; vacuolated cytoplasm; abundant intercellular mucoid matrix; gelatinous tumor; hemorrhagic changes; vascular tumor emboli in primary tumor; no mitotic figures in metastasis	NR	NR
39	Kamrin et al., 1964	10.1136/jnnp.27.2.157	NR	NR	Sacrum / sacrococcygeal	NR	NR	NR	Skin metastasis, exact cutaneous site not reported	Liver; lymph nodes; lung	NR	Skin metastasis reported in a patient with sacrococcygeal chordoma; patient also had liver, lymph node and lung metastases	NR	Indeterminate	NR	NR	NR	NR
40	Wang et al., 1968	10.1002/1097-0142(196807)22:1<162::aid-cncr2820220119>3.0.co;2-x	59	Male	Sacrum / sacrococcygeal	NR	~3.3	Multiple cutaneous sites	Buttock region; subcutaneous tissues; right axillary/infraclavicular regions; anterior right deltoid	Lung; pleura; pericardium; myocardium; retroperitoneum; liver; muscle; mediastinum	Other – multiple recurrent skin/subcutaneous nodules	Multiple recurrent skin nodules in the buttock region; later masses in the right axillary and infraclavicular regions and a large mass in the anterior right deltoid; autopsy showed extremely extensive subcutaneous nodules	NR	Multiple lesions	Skin nodules appeared approximately 2 years after external radiotherapy and approximately 3.3 years after initial diagnosis/admission; progressive widespread disease over the following years	Physaliphorous/vacuolated cells; mucinous matrix; polygonal cells; cords/chords of tumor cells; recurrent/metastatic chordoma histology	NR	NR
41	Persichetti et al., 2007	10.1111/j.1524-4725.2007.33329.x	61	Female	Sacrum / sacrococcygeal	NR	5	Surgical site / scar	Left coccyx / buttocks, just lateral to previous surgical scar	NR	Other – tender superficially eroded papule	Indurated, tender, superficially eroded 6-mm papule on the buttocks/left coccyx, just lateral to a well-healed scar from previous surgery	Tender	Solitary lesion	Present for approximately 2 months; developed 5 years after treatment of recurrent sacral/pelvic chordoma	Cords/nests of tumor cells; small ovoid cells; larger vacuolated cells; physaliphorous-like vacuolated cells; myxoid stroma; modest cytologic atypia	keratin; EMA; S100	CEA; Melan-A
42	Peramezza et al., 1993	10.1159/000247368	61	Male	Sacrum / sacrococcygeal	NR	0.5	Multiple cutaneous sites	Shoulders; left arm; face; head; trunk	No other metastases reported; liver-spleen scan, bone scan, abdominal and cranial CT showed no other metastatic sites	Nodule – skin-colored; Nodule – erythematous; Other – translucent/brownish-red/dark gray nodules	Numerous skin nodules ranging from several millimeters to several centimeters, especially on the head, face and trunk; translucent, often with telangiectasias, skin-colored, brownish-red or dark gray; elastic-hard, well-demarcated; facial disfigurement	Painful; Tender	Multiple lesions	Skin metastases appeared 6 months after removal of primary tumor; additional gradually growing lesions developed thereafter	Physaliphorous cells; myxoid/mucoid stroma; lobulated architecture; cords/nests of tumor cells; dermal and subcutaneous proliferation; central hemorrhages; peripheral pseudocapsule; vacuolated cytoplasm; polymorphous cells; few mitotic figures	cytokeratins; EMA (focal); S100 (focal); vimentin (focal)	NR
43	Unnikrishnan et al., 2019	https://ijbc.ir/browse.php?a_id=867&sid=1&slc_lang=en&ftxt=1	NR	NR	NR	NR	NR	NR	NR	NR	NR	NR	NR	NR	NR	NR	NR	NR
44	Gattoni et al., 2005	10.1016/s0151-9638(05)79335-2	56	Male	Skull base (clivus)	NR	6	Face	Left nasolabial groove / left cheek	Regional lymphadenopathy near the pharyngeal wall; recurrent nodular lesions of left upper lip and right cheek	Other – vegetating nodule	Nut-sized vegetating nodular lesion of the left nasolabial groove/left cheek, infiltrating deep planes and the orbicularis muscle of the upper lip	NR	Solitary lesion initially; later multiple nodular lesions	Appeared a few months after surgery for frontoglabellar relapse; primary skull-base chordoma developed 6 years earlier	Physaliphorous cells; myxoid stroma; cords/nests of tumor cells; anastomosing cords; PAS-positive cytoplasm; low pleomorphism; low mitotic index; no necrosis; no spindle cells; no glomus-like cells; dermal infiltration up to the dermoepidermal junction	vimentin; S100; EMA; cytokeratin 8; cytokeratin 19; high-molecular-weight cytokeratin (focal)	CEA; GFAP; HHF35 actin; desmin; cytokeratin 7
45	Benahmed et al., 2021	10.1016/j.jdcr.2021.10.020	88	Female	Sacrum / sacrococcygeal	NR	~8	Lower extremity	Right leg	No other metastases reported; whole-body and brain CT normal	Nodule – violaceous	Two dome-shaped, firm, bluish nodules on the right leg, painless, growing for 2 months; dermoscopy showed arborizing vessels and blue-gray structures	Asymptomatic; painless	Multiple lesions	2-month duration; appeared approximately 8 years after treatment of sacral chordoma	Physaliphorous cells; myxoid stroma; lobulated architecture; atypical cells; hyperchromatic cytoplasm; hypertrophic nuclei; pleomorphic cells	S100; pancytokeratin	NR
46	Suárez Conde et al., 2019	10.1016/j.jdcr.2019.07.029	60	Male	Sacrum / sacrococcygeal	NR	1	Multiple cutaneous sites	Gluteus; right arm; scalp; neck; face; back	Bone; lung; brain	Nodule – rose/pink	Multiple firm, pink, translucent, widely distributed nodules, mainly on the gluteus and right arm, followed by scalp, neck, face, and back; dermoscopy showed branched vessels on a yellow-white background	NR	Multiple lesions	Skin metastases developed approximately 1 year after initial diagnosis; progressive disease, later brain metastasis	Physaliphorous cells; myxoid/mucoid stroma; lobulated architecture; cords/nests of tumor cells; vacuolated cytoplasm; pleomorphic cells; PAS-positive cytoplasm; mild nuclear atypia/pleomorphism; fibrous septae	AE1/AE3; EMA	NR
47	Vergara et al., 2008	10.1007/s12094-008-0243-4	57	Male	Sacrum / sacrococcygeal	NR	8.3	Multiple cutaneous sites	Scalp; right arm; back; left arm; right shoulder; right gluteus	NR	Other – multiple subcutaneous metastases	Four scalp subcutaneous lesions initially removed; later additional cutaneous/subcutaneous lesions appeared on the right arm, back, scalp, left arm, right shoulder, right arm, right gluteus, and head	NR	Multiple lesions	Subcutaneous metastases appeared approximately 8 years after initial diagnosis and after repeated local recurrences	NR	NR	NR
48	Vergara et al., 2008	10.1007/s12094-008-0243-4	63	Male	Sacrum / sacrococcygeal	NR	~2.2	Multiple cutaneous sites	Neck; right arm; perianal region	Lung; bone / vertebral metastases including L3 and multiple vertebrae	Other – palpable subcutaneous metastases	Palpable subcutaneous metastases in the neck, right arm, and perianal region; biopsy confirmed metastatic chordoma	NR	Multiple lesions	Subcutaneous metastases appeared approximately 2 years and 2 months after diagnosis; shortly after local relapse and bone metastases	NR	NR	NR
49	Narang et al., 2018	https://pacificejournals.com/journal/index.php/apalm/article/view/1753	61	Male	Sacrum / sacrococcygeal	NR	~5	Multiple cutaneous sites	Forehead; lip; scalp	Bone; mediastinal lymph nodes; vertebrae; rib; pelvis	Other – multiple swellings mimicking neurofibromas	Multiple skin-covered swellings on forehead, lip and scalp, clinically diagnosed as neurofibromas; excised lesions measured 3 × 2 × 1 cm, 4 × 2 × 1.5 cm, and 7 × 5 × 3 cm; cut surface grey-tan to pale blue, with focal hemorrhage in scalp lesion	NR	Multiple lesions	Developed approximately 5 years after diagnosis of sacral chordoma, after multiple recurrences and systemic metastases	Physaliphorous cells; myxoid stroma; lobulated architecture; cords/nests of tumor cells; sheets of tumor cells; singly scattered tumor cells; vacuolated cytoplasm; eosinophilic cytoplasm; fibrous bands; mild to moderate nuclear atypia/pleomorphism; no mitotic activity; dermal involvement	pancytokeratin; vimentin	S100
50	Willis et al., 1930	10.1002/path.1700330418	41	Female	Sacrum / sacrococcygeal	NR	~4.2	Neck	Midline neck skin overlying the thyroid gland	Lung; bronchial/hilar lymph nodes; myocardium/heart; liver; spleen; kidneys; thyroid gland	Nodule – skin-colored	Smooth firm hemispherical cutaneous tumor, approximately 1–1.5 cm, in the midline skin of the neck overlying the thyroid	NR	Solitary lesion	Noticed approximately 4 months before death; disease duration from first tumor appearance was approximately 4.5 years	Physaliphorous cells; vacuolated cytoplasm; myxoid/mucinous stroma; abundant intra- and extracellular mucin; large multinucleated cells; polyhedral cells; low/absent mitotic activity; scant stroma; avascular tumor centers; sharply delimited expansive growth; focal calcification	NR	NR
51	Yarom et al., 1970	10.1002/1097-0142(197003)25:3<659::AID-CNCR2820250322>3.0.CO;2-Y	57	Male	Sacrum / sacrococcygeal	NR	~3	Multiple cutaneous sites	Lower abdomen; genitalia; right thigh	Skeletal muscle: left temporal muscle, left pectoralis major, right psoas; heart/myocardium	Other – multiple small round cutaneous elevations	Multiple small round cutaneous elevations, 0.1–2 cm, often with an umbilicated center, involving the lower abdomen, genitalia, and right thigh; on section, tumors were well-delineated, gray, firm, with gelatinous softening	NR	Multiple lesions	Cutaneous metastases appeared after local recurrence and approximately 3 years after initial diagnosis; total disease duration 9 years	Physaliphorous cells; vacuolated cytoplasm; myxoid/mucoid stroma; cords/clumps of tumor cells; polyhedral cells; low intercellular matrix in some areas; identical morphology in primary and metastatic sites	NR	NR
52	Wiacek et al., 2014	PMID: 24941302	NR	NR	NR	NR	NR	NR	NR	NR	NR	NR	NR	NR	NR	NR	NR	NR

Abbreviations: NR, not reported; IHC, immunohistochemistry; EMA, epithelial membrane antigen; CK, cytokeratin; INI1/SMARCB1, integrase interactor 1; TBXT, brachyury; FISH, fluorescence in situ hybridisation. Multi-case reports contribute one row per patient. The Kishimoto et al. (2012) imaging cohort (cases 31–37) comprised seven cutaneous/soft-tissue deposits without individual case-level data.

Table S2. JBI Critical Appraisal Checklist for Case Series applied to the five multi-patient series. Each series was independently appraised by two reviewers against the ten checklist items; the single-patient case reports were appraised separately with the JBI Critical Appraisal Checklist for Case Reports (Figure 7). No report was excluded on quality grounds.

Case series [ref]	Q1	Q2	Q3	Q4	Q5	Q6	Q7	Q8	Q9	Q10	"Yes" (n)
Su et al., 1993 [62]	Y	Y	Y	U	U	Y	Y	U	U	NA	5
Chambers & Schwinn, 1979 [67]	U	Y	Y	U	U	Y	Y	Y	U	NA	5
Vogin et al., 2016 [66]	Y	Y	Y	Y	U	Y	Y	Y	Y	U	8
Kishimoto et al., 2012 [68]	Y	Y	Y	U	U	U	U	Y	U	NA	4
Dimopoulos et al., 2023 [65]	Y	Y	Y	U	U	Y	Y	U	U	NA	5

Checklist items (JBI Critical Appraisal Checklist for Case Series): Q1, clear criteria for inclusion in the case series; Q2, condition measured in a standard, reliable way for all participants; Q3, valid methods used for identification of the condition for all participants; Q4, consecutive inclusion of participants; Q5, complete inclusion of participants; Q6, clear reporting of the demographics of the participants; Q7, clear reporting of clinical information of the participants; Q8, outcomes or follow-up results of cases clearly reported; Q9, clear reporting of the presenting site(s)/clinic(s) demographic information; Q10, statistical analysis appropriate.

Ratings: Y, yes; U, unclear; N, no; NA, not applicable. Cells are colour-coded (green, yes; amber, unclear; grey, not applicable). Where a series comprised very few patients (e.g., two cases), the items on consecutive and complete inclusion are of limited applicability. Items relating to treatment, post-intervention course and follow-up were more often rated "unclear," reflecting the diagnostic rather than therapeutic orientation of the source literature.

Table S3. Completed PRISMA 2020 checklist for this systematic review. Completed against the PRISMA 2020 statement (Page MJ, et al. BMJ 2021;372:n71); "Location reported" indicates the corresponding section, table or figure of the manuscript.

Section and Topic	Item #	Checklist item	Location reported
**TITLE**			
Title	1	Identify the report as a systematic review.	Title
**ABSTRACT**			
Abstract	2	See the PRISMA 2020 for Abstracts checklist.	Abstract
**INTRODUCTION**			
Rationale	3	Describe the rationale for the review in the context of existing knowledge.	Section 1
Objectives	4	Provide an explicit statement of the objective(s) or question(s) the review addresses.	Section 1 (final paragraph)
**METHODS**			
Eligibility criteria	5	Specify the inclusion and exclusion criteria and how studies were grouped for the syntheses.	Section 3.1
Information sources	6	Specify all databases, registers, websites, reference lists and other sources searched, and the date each was last searched.	Section 3.1 (PubMed/MEDLINE, inception–June 2025; reference-list screening)
Search strategy	7	Present the full search strategies for all databases, registers and websites, including filters and limits.	Section 3.1 (search string reported)
Selection process	8	Specify methods used to decide whether a study met inclusion criteria, how many reviewers screened, and whether independently.	Section 3.1 (two reviewers, independent, consensus)
Data collection process	9	Specify methods used to collect data from reports, number of reviewers, independence, and processes to confirm data with investigators.	Section 3.2 (two reviewers; piloted standardised form)
Data items	10a	List and define all outcomes for which data were sought.	Sections 3.2–3.3 (pooled prevalences; latency time-to-event; brachyury uptake over time)
	10b	List and define all other variables; describe any assumptions about missing or unclear information.	Section 3.2 (variables listed; available-case analysis, no imputation)
Study risk of bias assessment	11	Specify methods used to assess risk of bias, the tool(s) used, number of reviewers and independence.	Section 3.4 (JBI Case Reports and Case Series checklists; Murad framework)
Effect measures	12	Specify the effect measure(s) used in the synthesis or presentation of results.	Section 3.3 (pooled prevalence; hazard ratio; correlation)
Synthesis methods	13a	Describe the processes used to decide which	Sections 3.2–3.3 (one-stage individual-

		studies were eligible for each synthesis.	participant-data synthesis)
	13b	Describe any methods required to prepare the data for presentation or synthesis.	Section 3.2 (per-variable available-case analysis; explicit denominators; no imputation)
	13c	Describe any methods used to tabulate or visually display results of individual studies and syntheses.	Section 3.3; Tables 2–3; Figures 2–6; Supplementary Table S1
	13d	Describe any methods used to synthesise results and the rationale; for meta-analysis, describe the model(s), heterogeneity method(s) and software.	Section 3.3 (logistic-normal random-effects; Cochran Q, I^2, τ^2; one-stage Cox with robust SE; Python — SciPy, statsmodels, lifelines)
	13e	Describe any methods used to explore possible causes of heterogeneity.	Section 3.3 (age subgroup; meta-regression of brachyury use on publication year)
	13f	Describe any sensitivity analyses conducted.	Section 3.3 (Freeman–Tukey double-arcsine sensitivity analysis)
Reporting bias assessment	14	Describe any methods used to assess risk of bias due to missing results within a synthesis.	Sections 3.1, 3.4 and 6.6 (language/publication bias considered)
Certainty assessment	15	Describe any methods used to assess certainty in the body of evidence.	Sections 3.3 and 6.6 (formal GRADE not applied; interpreted as hypothesis-generating)
**RESULTS**			
Study selection	16a	Describe the results of the search and selection process, ideally using a flow diagram.	Section 4.1; Figure 1 (PRISMA flow diagram)
	16b	Cite studies that appeared to meet the inclusion criteria but were excluded, and explain why.	Section 4.1 (record counts and reasons for exclusion)
Study characteristics	17	Cite each included study and present its characteristics.	Sections 4.2–4.6; Table 3; Supplementary Table S1
Risk of bias in studies	18	Present assessments of risk of bias for each included study.	Section 4.7; Figure 7; Supplementary Table S2
Results of individual studies	19	Present, for each study, summary data and an effect estimate with precision.	Table 3; Supplementary Table S1
Results of syntheses	20a	Summarise the characteristics and risk of bias among contributing studies for each synthesis.	Sections 4.6–4.7
	20b	Present results of all statistical syntheses; for meta-analysis, give the summary estimate, precision and heterogeneity.	Section 4.6 (pooled prevalences with 95% CI; I^2 = 0%); Figure 6
	20c	Present results of all investigations of possible causes of heterogeneity.	Sections 4.4–4.5 (age subgroup; brachyury meta-regression); Figures 4–5
	20d	Present results of all sensitivity analyses.	Section 4.6 and Section 3.3 (Freeman–Tukey vs logistic-normal)

Reporting biases	21	Present assessments of risk of bias due to missing results for each synthesis.	Section 6.6 (limitations: incomplete reporting; language/publication bias)
Certainty of evidence	22	Present assessments of certainty in the body of evidence for each outcome.	Sections 4.6 and 6.6 (estimates framed as a quantified consolidation; hypothesis-generating)
**DISCUSSION**			
Discussion	23a	Provide a general interpretation of the results in the context of other evidence.	Section 6 (6.1–6.5)
	23b	Discuss any limitations of the evidence included in the review.	Section 6.6
	23c	Discuss any limitations of the review processes used.	Section 6.6 (IPD from published reports; does not meet full PRISMA-IPD standard)
	23d	Discuss implications of the results for practice, policy and future research.	Sections 6.5 and 7
**OTHER INFORMATION**			
Registration and protocol	24a	Provide registration information, or state that the review was not registered.	Section 3.1 (not registered)
	24b	Indicate where the protocol can be accessed, or state that one was not prepared.	Section 3.1 (protocol not prospectively registered)
	24c	Describe and explain any amendments to registration or protocol information.	Not applicable
Support	25	Describe sources of financial or non-financial support and the role of funders.	Funding; Acknowledgments (APC funded; funder had no role)
Competing interests	26	Declare any competing interests of the review authors.	Conflicts of Interest statement
Availability of data, code and other materials	27	Report which materials are publicly available and where (data collection forms, extracted data, analytic code, other materials).	Data Availability Statement; extracted data in Supplementary Table S1; analysis code (Python) available from the corresponding author on request

Abbreviations: CI, confidence interval; GRADE, Grading of Recommendations Assessment, Development and Evaluation; IPD, individual-participant data; JBI, Joanna Briggs Institute; SE, standard error.
